# Caffeine Intake and Performance Outcomes in Taekwondo Athletes: A Systematic Review and Meta-Analysis

**DOI:** 10.3390/metabo16100784

**Published:** 2026-10-16

**Authors:** Limin Cai, Jintao Guo, Si Chen

**Affiliations:** 1 Department of Physical Education, North China Electric Power University, Beijing 102206, China; 2 School of Medicine and Health Care, Shandong Yingcai University, Jinan 250104, China; 3 College of Physical Education and Health Sciences, Zhejiang Normal University, Jinhua 321000, China

**Keywords:** caffeine, taekwondo, athletic performance, meta-analysis, nutritional supplementation

## Abstract

**Background:** The ergogenic role of caffeine in taekwondo-specific performance has not been comprehensively synthesized. This study aimed to evaluate the effects of caffeine supplementation on performance outcomes in taekwondo athletes. **Methods:** PubMed, Web of Science, Cochrane Library, and SPORTDiscus were searched up to April 2026. Randomized controlled trials comparing caffeine supplementation with placebo in taekwondo athletes were included, including both acute and longer/repeated intervention protocols where eligible. Effect sizes were pooled as Hedges’ g, a small-sample-corrected standardized mean difference, using random-effects models. Evidence certainty was assessed using the GRADE approach. **Results:** Caffeine supplementation was associated with favorable pooled effects for explosive performance (Hedges’ g = 0.40; 95% CI 0.07–0.72; moderate-certainty evidence), reaction agility (g = 1.43; 95% CI 0.79–2.08; low-certainty evidence), technical accuracy (g = 1.05; 95% CI 0.32–1.77; low-certainty evidence), FSKT-10s performance (g = 3.31; 95% CI 1.42–5.19; very-low-certainty evidence), and FSKT-mult performance (g = 4.29; 95% CI 1.88–6.70; very-low-certainty evidence). No significant pooled effect was observed for endurance performance (g = 0.10; 95% CI −0.19–0.39; low-certainty evidence). **Conclusions:** Across the clinically heterogeneous acute and longer/repeated protocols included in this review, caffeine supplementation was associated with favorable pooled effects for explosive performance, reaction agility, and technical accuracy, whereas no significant pooled effect was observed for endurance outcomes. These estimates represent averages across different intervention protocols and should not be interpreted as a uniform effect of either acute or repeated supplementation. FSKT-based effects were large but should be interpreted cautiously given high heterogeneity. Observed dose-related patterns should be considered hypothesis-generating because formal subgroup or dose–response analyses were not conducted; independent replication is needed.

## 1. Introduction

Caffeine is one of the most commonly used nutritional supplements worldwide with well-established ergogenic effects. It enhances exercise performance through antagonizing adenosine receptors, promoting calcium ion release, enhancing neuromuscular excitability, and increasing central nervous system activation [1]. Extensive research demonstrates that caffeine produces ergogenic effects across various exercise modalities, including strength, endurance, and power [2,3]. However, significant variations exist in its effects across different sports, dosages, and ingestion strategies. Particularly in high-intensity intermittent combat sports, the mechanisms and manifestations of caffeine’s effects remain controversial [4].

Critically, existing systematic reviews aggregate heterogeneous combat sports (judo, boxing, wrestling, MMA) without accounting for sport-specific biomechanical and metabolic demands [5]. This “one-size-fits-all” approach obscures crucial within-sport variations in technique complexity, scoring systems, and physical demands that may differentially modulate caffeine’s ergogenic mechanisms. For instance, a recent meta-analysis in volleyball demonstrated that caffeine’s physical performance benefits (jumping: g = 0.24; strength: g = 0.31) effectively translated into improved sport-specific skill execution (g = 0.52), including serve and attack accuracy [6]. This underscores the necessity of sport-specific investigations to understand how caffeine affects the unique demands of each sport [5,7,8]. Additionally, moderate-to-high doses of caffeine significantly improve mean and peak barbell velocity in bench press and rowing movements, and enhance judogi grip strength performance [9].

Systematic reviews demonstrate that acute ingestion of 3–6 mg/kg caffeine effectively improves peak power, muscular endurance, reaction speed, and technical execution stability [5,7,8]. Observed dose-related patterns should be considered hypothesis-generating because no formal subgroup or dose–response meta-analysis was conducted [8,10,11,12,13]; in boxing and mixed martial arts, caffeine prolongs high-intensity offensive-defensive duration and improves muscular responses [13,14,15]; in wrestling, divided low-dose strategies prove superior to single high-dose approaches [16,17]. Furthermore, caffeine combined with sodium bicarbonate, music stimulation, or post-activation performance enhancement (PAPE) demonstrates synergistic ergogenic effects [6,18,19], Despite this body of evidence, taekwondo presents a unique physiological profile that warrants dedicated investigation. Unlike grappling sports (judo, wrestling, Brazilian jiu-jitsu) where continuous muscle tension and isometric strength dominate [10,11,12,13,20], or striking sports with longer combat durations (boxing, MMA) [14,15,17], taekwondo is characterized by: (1) higher kicking-to-punching ratios (>80% vs. <40% in karate); (2) explosive lower-limb movements requiring peak velocities >15 m/s; (3) reactive agility demands under opponent-initiated constraints; and (4) technical scoring systems rewarding precision over accumulated damage. These sport-specific attributes engage distinct neuromuscular pathways—particularly fast-twitch fiber recruitment and stretch-shortening cycle efficiency—where caffeine’s adenosine antagonism may produce divergent effects compared to upper-body dominant or grappling-based sports [1].

Recent evidence indicates that caffeine’s influence extends beyond metabolic levels, also improving performance by enhancing neuromuscular efficiency and cognitive processing capacity. Systematic reviews reveal that caffeine expectancy effects can produce impacts comparable to pharmacological effects across various exercise tasks [21]. For instance, 6 mg/kg caffeine significantly improves peak power and neuromuscular efficiency in boxers [15]; in taekwondo athletes, 200 mg caffeine ingestion enhances Wingate peak power and executive control [22]. Caffeine combined with PAPE further elevates anaerobic power and muscular output within short timeframes [19].

However, systematic research specifically targeting taekwondo remains limited. Although existing literature has validated caffeine’s ergogenic effects in other combat sports, comprehensive evidence regarding its integrated impact on technical success rate, rhythm control, and cognitive responses in taekwondo is lacking. Moreover, caffeine’s effects are significantly influenced by individual differences, with habitual intake, training level, and sex all modulating effect magnitude [9,13,23]. Caffeine supplementation at dosages typically employed to enhance exercise performance (3–9 mg/kg) may elicit a range of side effects in certain individuals. These effects can include tachycardia, palpitations, nervousness, gastrointestinal discomfort, headaches, and sleep disturbances. The individual responses and the severity of these side effects are often dose-dependent [24].

Some studies suggest that in technically dominated sports involving complex movements, caffeine’s direct effects may be relatively weak, while its indirect effects through reducing perceived fatigue and enhancing cognitive control are more prominent [25].

Unlike previous systematic reviews and network meta-analyses that combined heterogeneous combat or intermittent sports, this meta-analysis focuses specifically on taekwondo athletes. This approach allows us to address unresolved questions, including whether caffeine has greater effects on technical performance and reaction agility than on endurance-related outcomes in taekwondo.

In this review, evidence from other combat sports is used only to provide background rationale, whereas the quantitative synthesis is restricted to studies involving taekwondo athletes. The purpose of this systematic review and meta-analysis was to systematically synthesize the available evidence on the effects of caffeine supplementation on taekwondo-specific performance outcomes. Specifically, this study aimed to quantify caffeine’s effects across different performance domains, including reaction agility, endurance-related outcomes, explosive performance, and taekwondo-specific technical performance. In addition, this review sought to descriptively explore whether patterns of response may vary according to caffeine dose, age, and training experience. These exploratory analyses were intended to generate hypotheses for future research rather than to provide confirmatory subgroup evidence.

## 2. Materials and Methods

This systematic review and meta-analysis was conducted following the Preferred Reporting Items for Systematic Reviews and Meta-Analyses (PRISMA 2020) [12,26] and was registered in PROSPERO (registration number: CRD420251150455), an international prospective register of systematic reviews. The PROSPERO registration date was [18 September 2025]. The registration was completed before formal data extraction and quantitative synthesis.

### 2.1. Literature Search

Two authors independently searched the PubMed, Web of Science, SPORTDiscus, and Cochrane Library databases from inception to April 2026. Studies were retrieved from electronic databases using database-specific Boolean search strategies combining taekwondo- or combat-sport-related terms, caffeine-related terms, and performance-related terms. The exact search strings for each database, applied filters, and the number of records retrieved are reported in Supplementary Table S1. Additionally, reference lists of eligible included studies were manually searched. The search was limited to English-language publications. Any disagreements were resolved through discussion between the two reviewers; when consensus could not be reached, a third reviewer was consulted.

To assess whether the main strategy missed relevant studies, we additionally conducted a supplementary sensitivity search in April 2026 using broader related terms, including coffee, methylxanthine, supplementation, ergogenic aid, combat sport, FSKT, and “Frequency Speed of Kick Test.” The database-specific supplementary search strings, applied filters, and number of records retrieved are reported in Supplementary Table S1. This supplementary search was used only to verify search sensitivity and did not identify additional eligible taekwondo caffeine intervention studies beyond those included in the main analysis.

### 2.2. Literature Search Inclusion and Exclusion Criteria

Inclusion and exclusion criteria were defined using the PICO framework (Table 1). Eligible studies (1) evaluated the effects of caffeine ingestion on sport-specific performance in taekwondo athletes of all competitive levels, with no restrictions on age or sex; (2) adopted randomized controlled trial (RCT) or randomized crossover trial designs; (3) administered caffeine as a standalone intervention or combined with other modalities, with no restrictions on dosage, timing, or mode of administration; (4) included a placebo control condition with identical taekwondo training content, differing only by replacement of caffeine with placebo; and (5) reported quantitative taekwondo performance outcomes with sufficient data for meta-analysis (means, standard deviations, and sample sizes). Outcomes were synthesized into two overarching domains consistent with the Results: physical ability (reaction agility, endurance, and explosive performance) and technical performance (FSKT-10s, FSKT-mult, and technical accuracy). Finally, (6) only peer-reviewed published articles were included.

Studies in which caffeine was combined with another modality were eligible only when the comparison condition received the identical co-intervention and differed solely in the provision of caffeine versus placebo. For multi-arm, factorial, or crossover trials, only a matched caffeine–placebo comparison was extracted. Comparisons in which music, conditioning activity, nutritional supplementation, warm-up procedures, or other non-caffeine components differed between the intervention and comparator conditions were not included.

Exclusion criteria: (1) Review articles; (2) Systematic reviews and meta-analyses; (3) Conference abstracts; (4) Conference proceedings; (5) Articles without specific research outcome data; (6) Non-randomized controlled trial designs; (7) Observational studies, case reports, or case series; (8) Studies lacking statistical parameters required for meta-analysis; (9) Non-taekwondo-specific athletes, including athletes from other martial arts, mixed martial arts fighters, or general populations. Although non-taekwondo combat-sport samples were excluded, studies were retained when the original article explicitly reported that participants had a taekwondo training background and provided extractable outcomes relevant to taekwondo-specific or kicking performance.

Eligible interventions included acute caffeine administration as well as longer or repeated caffeine supplementation protocols when caffeine was compared with placebo under otherwise similar taekwondo training or testing conditions. Therefore, the pooled analyses should be interpreted as overall effects of caffeine supplementation rather than exclusively acute single-dose effects.

### 2.3. Data Extraction

The primary data for this study were outcome measures of taekwondo athletic performance-related indicators assessed in the included studies. Other relevant data extracted included study reference, study design, participant characteristics (sample size, age, gender distribution, competitive level, training experience, physical condition), intervention details (caffeine dose, timing of administration, administration method), control conditions (placebo type, administration method), outcome assessment methods (technical performance, strength, speed, endurance, agility, reaction agility and other taekwondo-specific tests), and primary study results (statistical parameters such as means, standard deviations, sample sizes). Because several eligible reports originated from closely affiliated research teams, we additionally compared publications with respect to recruitment setting and period, sample size, age and sex distribution, competitive level, eligibility criteria, ethics or registration information, and any available cohort or participant identifiers to assess possible duplication or participant overlap. Shared authorship, study setting, ethics approval, or similarity of experimental protocols alone was not considered evidence of participant overlap; overlap was considered supported only when explicitly reported or otherwise demonstrable from the available study information.

If outcome data were missing, corresponding authors were contacted by email. A follow-up email was sent after two weeks. Studies were excluded only when the required quantitative data could not be obtained from the article, Supplementary Materials, or author correspondence.

Outcome domains were operationally defined before quantitative synthesis. Reaction agility referred to tests assessing stimulus-response speed, kicking reaction time, or taekwondo-specific agility performance. Endurance performance referred to fatigue-related or aerobic/anaerobic endurance indicators, such as time to exhaustion, VO_2_max, ventilatory threshold, or fatigue index. Explosive performance referred to short-duration power or jump-related outcomes, such as peak power, relative power, or vertical jump performance. Technical accuracy referred to taekwondo-specific skill execution outcomes, such as successful kick percentage, attack accuracy, competition score, or other accuracy-based technical indicators. FSKT-10s and FSKT-mult were analyzed as separate taekwondo-specific technical performance outcomes because they represent standardized kicking-frequency tests.

These outcome categories were defined as broad functional domains rather than sets of identical measurement instruments. Different tests were pooled using standardized mean differences only when they were judged to assess the same underlying functional construct [27]. Differences in task complexity, neuromuscular demand, and cognitive involvement were considered potential sources of heterogeneity. Consequently, pooled estimates should be interpreted as average effects at the functional-domain level rather than effects on individual tests, as standardization does not eliminate conceptual heterogeneity. The exact outcomes and their study-level assignments are reported in Supplementary Table S4.

### 2.4. Methodological Quality Assessment and Summary of Evidence

The methodological quality of the included trials was independently assessed by two reviewers (LC and WB) using the PEDro scale. The 11 items were rated as yes (1) or no (0); item 1, eligibility criteria, was recorded but was not included in the total score, which therefore ranged from 0 to 10. Ratings were based solely on information explicitly reported in the main article or its Supplementary Materials. Items that were not reported or were insufficiently described were scored as no [27]. For crossover trials, random allocation referred to randomization of the order in which the intervention conditions were received. Allocation concealment was rated as present only when the allocation or treatment-order sequence was protected from the person determining eligibility or enrolment. Blinding of intervention personnel referred to the person administering the caffeine or placebo condition, whereas assessor blinding required explicit blinding of the person measuring at least one key outcome. Adequate follow-up required outcome data for more than 85% of the initially randomized participants for at least one key outcome. Intention-to-treat was rated as present when participants were analyzed according to their randomized assignment or treatment order, or when the report explicitly confirmed that all participants received the assigned conditions.

PEDro total scores were reported descriptively and were not interpreted as overall low-, moderate-, or high-risk-of-bias categories. Disagreements were resolved through discussion with a third reviewer (SC). Study-by-study item-level ratings and the supporting information for the key methodological items are presented in Supplementary Table S3.

The certainty of evidence for each outcome was assessed using the GRADE framework across five domains: risk of bias, inconsistency, indirectness, imprecision, and publication bias. Judgements were made separately for each outcome and were based on the studies contributing to that specific meta-analysis. Participant numbers represented unique randomized participants; participants in crossover trials were counted once rather than once per intervention condition [28]. Imprecision was judged primarily according to the width of the 95% confidence interval in relation to no effect and plausible meaningful effects, with the unique participant sample size and optimal information size considered as supporting criteria. Differences in outcome tests, caffeine doses, intervention durations, and co-interventions were considered under inconsistency or indirectness without double-counting the same limitation. Because fewer than 10 studies contributed to each outcome, funnel plots were considered exploratory and were not used to infer the absence of publication bias.

### 2.5. Statistical Analysis

Several included studies employed randomized crossover designs in which the same participants completed both caffeine and placebo conditions. Study-level standardized mean differences were calculated from the between-condition mean difference divided by the pooled condition-specific standard deviation, followed by a small-sample correction to obtain Hedges’ g. The main random-effects estimates were calculated using condition-level sampling variances under an independence assumption. Because this approach does not account for the within-participant dependence inherent to crossover designs, additional paired sensitivity analyses were performed [29,30].

When the standard deviation of the paired differences or the within-participant correlation was unavailable, the latter was imputed at r = 0.30, 0.50, and 0.70. For each assumed correlation, the variance of the within-participant difference was approximated as SD_diff^2^ = SD_C^2^ + SD_P^2^ − 2r × SD_C × SD_P, where SD_C and SD_P represent the condition-specific standard deviations. The correlation-adjusted effect-size variances were used in generic inverse-variance random-effects models. The assumed correlation affected the sampling variances, standard errors, and study weights, potentially changing the pooled estimates without directly altering the study-level standardized mean differences. These sensitivity analyses are reported in Supplementary Table S2.

Data extraction and statistical analyses were performed independently by two researchers. Effect sizes were expressed as Hedges’ g with 95% confidence intervals. The small-sample correction specified for the analysis was g = d × [1 − 3/{4(n_C + n_P) − 9}], where d represents the difference between the caffeine and placebo means divided by the pooled condition-specific standard deviation, and n_C and n_P denote the numbers of observations under each condition [31]. For crossover studies with n paired participants, n_C = n_P = n refers to measurements obtained from the same individuals rather than two independent participant groups. The same study-level effect-size correction was retained across the correlation-adjusted sensitivity analyses.

We also examined whether the broad domains could be stratified according to the exact test instrument or test type. However, after stratification, most instrument-specific categories contained only one or two independent studies, which was considered insufficient for an informative quantitative subgroup synthesis; therefore, these test-level distinctions were retained descriptively in Supplementary Table S4 rather than pooled separately.

A random-effects model (DerSimonian-Laird method) was employed for meta-analysis to account for between-study heterogeneity [32]. Heterogeneity was assessed using the I^2^ statistic, where I^2^ < 25% indicated low heterogeneity, 25–50% indicated moderate heterogeneity, and >50% indicated substantial heterogeneity [33].

Prespecified subgroup meta-analyses were not conducted. This decision was made because the number of included studies per outcome was small, which would have provided insufficient statistical power for reliable subgroup comparisons. In addition, potential effect modifiers, including caffeine dose, sex, age, training experience, and co-intervention strategies, were not independently distributed across studies and were often intertwined. Under these conditions, subgroup analyses could have generated spurious or overinterpreted results. Accordingly, any dose-, age-, sex-, or training-status-related patterns discussed below were based solely on post hoc exploratory descriptive comparisons across studies; no formal subgroup interaction tests were performed, and these patterns were not interpreted as independent effect-modifier effects [27,29].

Leave-one-out analyses were additionally used to assess whether any pooled result was disproportionately influenced by a single study. Given the small number of studies and very high heterogeneity in the FSKT analyses, an additional sensitivity analysis was performed using restricted maximum likelihood (REML) estimation with Hartung–Knapp-adjusted confidence intervals. To further assess whether the exceptionally large FSKT estimates were disproportionately influenced by a single extreme study, a post hoc influence analysis was performed by excluding the study with the largest study-level Hedges’ g from each FSKT meta-analysis and re-estimating the pooled effect using the primary random-effects model.

To avoid double-counting and preserve independence of effect sizes, each study contributed only one effect size to each outcome-domain meta-analysis. When a study reported multiple outcomes within the same domain, the most directly relevant prespecified outcome was selected. When multiple closely related measures reflected the same construct and selection of a single measure was not appropriate, they were combined into one study-level effect size before inclusion in the meta-analysis. For time-based outcomes, the direction of effect was reversed when lower values indicated better performance, so that positive Hedges’ g values consistently favored caffeine supplementation.

The primary analyses combined acute and longer-term or repeated caffeine intervention protocols. Because the number of studies within each outcome and intervention category was insufficient for reliable subgroup analysis, the pooled estimates were interpreted as the overall effects of caffeine supplementation rather than as effects specific to either intervention type.

## 3. Results

### 3.1. Study Selection

**Figure 1:**
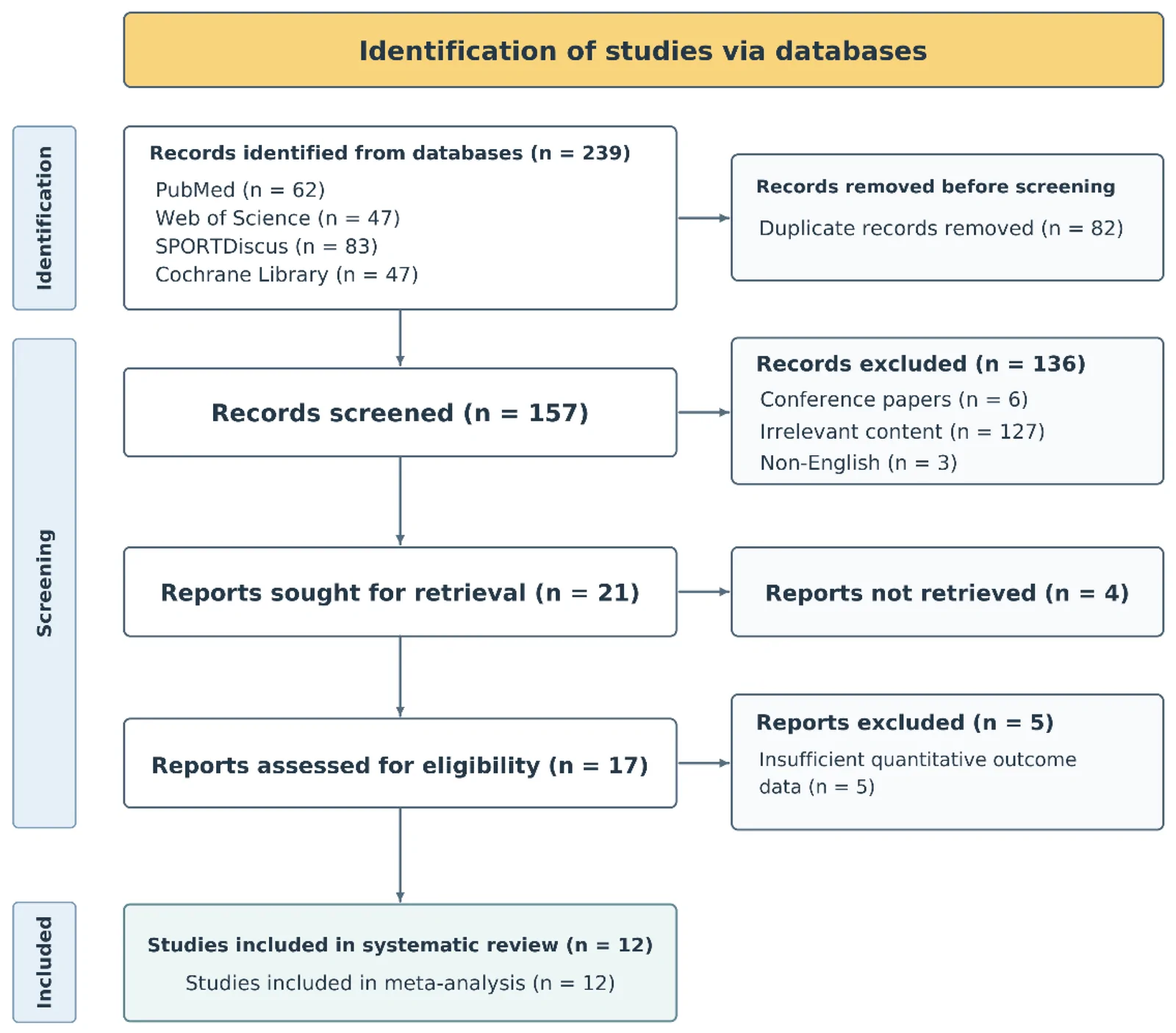
PRISMA flowchart.

Figure 1 presents the PRISMA flow diagram of the study-selection process. A total of 239 records were identified through searches of four databases (PubMed, *n* = 62; Web of Science, *n* = 47; SPORTDiscus, *n* = 83; and Cochrane Library, *n* = 47). After removal of 82 duplicate records, 157 records underwent title and abstract screening, of which 136 were excluded. Twenty-one reports were sought for retrieval; four reports could not be retrieved. Consequently, 17 full-text reports were assessed for eligibility. Five reports were excluded because the quantitative outcome data required for meta-analysis were unavailable. Finally, 12 studies [1,23,31,32,33,34,35,36,37,38,39,40] were included in the meta-analysis.

### 3.2. Characteristics of Included Studies

This systematic review and meta-analysis ultimately included 12 randomized controlled trials involving 205 taekwondo athletes. The publication years of the studies ranged from 2014 to 2026, with sample sizes ranging from 10 to 52 participants [1,23,34,35,36,37,38,39,40,41,42,43].

Regarding participant characteristics, the age distribution presented two main groups: six studies focused on adolescent athletes (≤18 years) with a mean age of 17–18 years, while six studies concentrated on adult athletes (>18 years) with an age range of 18–26 years. In terms of gender distribution, seven studies included only male athletes, three studies specifically recruited female athletes, and two studies included both male and female participants. Training experience varied considerably, with three studies involving participants with ≤5 years of experience, two studies with 6–7 years, and five studies with ≥7 years, reflecting broad representation across different levels from amateur to elite athletes.

Concerning intervention design, caffeine dosage exhibited a three-tiered stratification pattern. The low-dose group (≤3 mg/kg) comprised six studies, primarily using oral aqueous solutions; the moderate-dose group (5 mg/kg) encompassed two studies, both utilizing caffeine capsules; the high-dose group (6 mg/kg) included three studies employing capsules and oral rinsing methods, respectively. Additionally, one study used a fixed dose of 200 mg oral aqueous solution. Regarding study design, seven studies adopted acute administration protocols to assess immediate effects, whereas five studies involved longer or repeated intervention protocols. Accordingly, the pooled analyses were interpreted as overall effects of caffeine supplementation across eligible protocols rather than as exclusively acute single-dose effects.

In terms of outcome measures, the taekwondo-specific performance indicators assessed could be categorized into four main domains. Technical performance tests were reported in seven studies, primarily including the Taekwondo-Specific Agility Test (TSAT), Frequency Speed of Kick Test (FSKT-10s and FSKT-mult), and percentage of successful kicks. Reactive agility indicators were measured in eight studies, covering reaction agility, kicking time, and agility course tests. Endurance performance assessment appeared in four studies, encompassing aerobic capacity indicators such as time to exhaustion, maximal oxygen uptake (VO_2_max), and ventilatory threshold. Explosive performance tests were implemented in two studies, primarily measuring peak power, vertical jump height, and power-to-weight ratio.

Regarding safety, 7 of 12 studies reported no adverse events, 3 reported mild symptoms (gastrointestinal discomfort, headaches, tachycardia), and 2 did not report safety data (see Table 2). Due to inconsistent reporting, systematic meta-analysis of adverse events was not conducted.

Although some included studies employed general physiological tests (e.g., Wingate or VO_2_max), all participants were exclusively taekwondo athletes, and no samples from other combat sports or non-taekwondo populations were included.

A considerable proportion of the included evidence originated from closely affiliated research teams using similar participant populations and experimental protocols. We therefore cross-checked the original reports with respect to recruitment setting and period, sample size, age and sex distribution, competitive level, eligibility criteria, ethics or registration information, and any available cohort identifiers. No original publication explicitly reported reuse of the same participant cohort, and no direct evidence of overlapping participant samples or duplicate reporting was identified from the information available in the published reports. However, participant-level identifiers and complete recruitment-period information were not available for all studies; therefore, participant-level non-overlap across publications could not be independently verified with certainty [37,38,39,40,42]. The characteristics of the included studies are summarized in Table 2.

**Table 2:** Characteristics of the included trials examining the effects of caffeine supplementation on performance in taekwondo athletes.

Study	Sample Size	Age	Sex (Men/Women)	Intervention	Dosage Form	Training Experience	Caffeine Dose	Outcome Measures	Side Effects
**Delleli et al. 2023** [39]	16	18.25 ± 0.75	16/0	Caffeine + Taekwondo training	Oral aqueous solution	≥8 years	3 mg/kg	TSAT ↑; FSKT-10s ↑; FSKT-mult ↑	Mild gastrointestinal diarrhea
				Placebo + Taekwondo training					
**Delleli et al. 2024 [40]**	16	17.69 ± 0.60	0/16	Caffeine + Taekwondo training	Oral aqueous solution	≥8 years	3 mg/kg	Jump Time ↑	no report
				Placebo + Taekwondo training					
**Delleli et al. 2024b [43]**	16	17–19 years	0/16	Caffeine + Taekwondo training	Oral aqueous solution	≥8 years	3 mg/kg	TSAT ↑; FSKT-10s ↑; FSKT-mult ↑	A few athletes reported moderate headaches and diarrhea
				Placebo + Taekwondo training					
**Lopes-Silva et al. 2015** **[34]**	10	21 ± 4	10/0	Caffeine + Taekwondo training	Oral aqueous solution	≥9 years	5 mg/kg	Number of Attacks →; Attack Time (s) →; Peak blood lactate ↑	Mainly for the sense of energy and tachycardia
				Placebo + Taekwondo training					
**Nana et al. 2025 [42]**	13	18–25 years	13/0	Caffeine + Taekwondo training	Caffeine capsules	2–5 years	3 mg/kg	reaction agility →; Road Agility Test →	no report
				Placebo + Taekwondo training					
**Sun et al. 2022 [23]**	10	26 ± 2.4	6/4	Caffeine + Taekwondo training	Oral aqueous solution	6 ± 1 years	200 mg	Peak Power (W) ↑; Peak Power/Body Mass (W/kg) ↑;Average Power/Body Mass (W/kg) ↑; Average Score in Simulated Competition →; Winning Rate →; Average Power →;Fatigue Index →; Time to Peak Power →	no report
				Placebo + Taekwondo training					
**Saremi et al. 2024 [41]**	12	24 ± 2	12/0	Caffeine + Taekwondo training	Caffeine capsules	≥3 years	6 mg/kg	Time to Exhaustion ↑; Vertical Jump Height ↑; VO_2_max →; Oxygen Uptake at VT2 →; VT2 →; Rating of Perceived Exertion →; Relative Average Power →; Wingate Fatigue Index →	no report
				Placebo + Taekwondo training					
**Santos et al. 2014 [1]**	10	24.9 ± 7.3 years	10/0	Caffeine + Taekwondo training	Caffeine capsules	≥7 years	5 mg/kg	reaction agility ↑; Kick Time →; Subjective Physical Sensation Score →	no report
				Placebo + Taekwondo training					
**Ouergui et al. 2022 [37]**	20	17.5 ± 0.7	10/10	Caffeine + Taekwondo training	Oral aqueous solution	≥6 years	3 mg/kg	TSAT ↑; FSKT-10s ↑; FSKT-mult ↑	no report
				Placebo + Taekwondo training		≥6 years			
**Ouergui et al. 2023 [38]**	52	17.8 years	26/26	Caffeine + Taekwondo training	Oral aqueous solution	no report	3 mg/kg	TSAT ↑; FSKT-10s ↑; FSKT-mult ↑	no report
				Placebo + Taekwondo training					
**Pak et al. 2020 [36]**	27	17 ± 3	18/9	Caffeine + Taekwondo training	Oral gargling	no report	6 mg/kg	Successful kick percentage ↑	no report
				Placebo + Taekwondo training					
**Cortez et al. 2017 [35]**	13	22.8	no report	Caffeine + Taekwondo training	Oral aqueous solution	3.7 ± 1.8	5 mg/kg	Rectus Femoris reaction agility ↑	no report
				Placebo + Taekwondo training					

Note: ↑ indicates a statistically significant improvement or increase in the caffeine condition compared with placebo; → indicates no statistically significant difference between the caffeine and placebo conditions.

### 3.3. Quality Assessment of Included Studies

Following verification against the original trial reports, the revised study-by-study PEDro ratings are presented in Supplementary Table S3. Item 1 (eligibility criteria) was excluded from the calculation of the total score, and methodological items that were not reported or were insufficiently described were scored as no. Consequently, no included study received a PEDro score of 10/10.

The maximum PEDro score was 9/10: six studies (50.0%) scored 9, four (33.3%) scored 8, and two (16.7%) scored 7 (Supplementary Table S3). Thus, the observed PEDro scores ranged from 7 to 9. At the item level, 100% (12/12) of the studies met the allocation-concealment criterion, 91.7% (11/12) achieved investigator blinding, and 33.3% (4/12) achieved assessor blinding. Notably, 91.7% (11/12) of the studies achieved participant blinding; however, due to the inherent nature of caffeine supplementation—whereby participants may perceive the physiological stimulatory effects of caffeine—this represents an inherent limitation of such intervention studies.

### 3.4. Meta-Analysis and Sensitivity Analyses

#### 3.4.1. Meta-Analysis of Results

**Figure 2:**
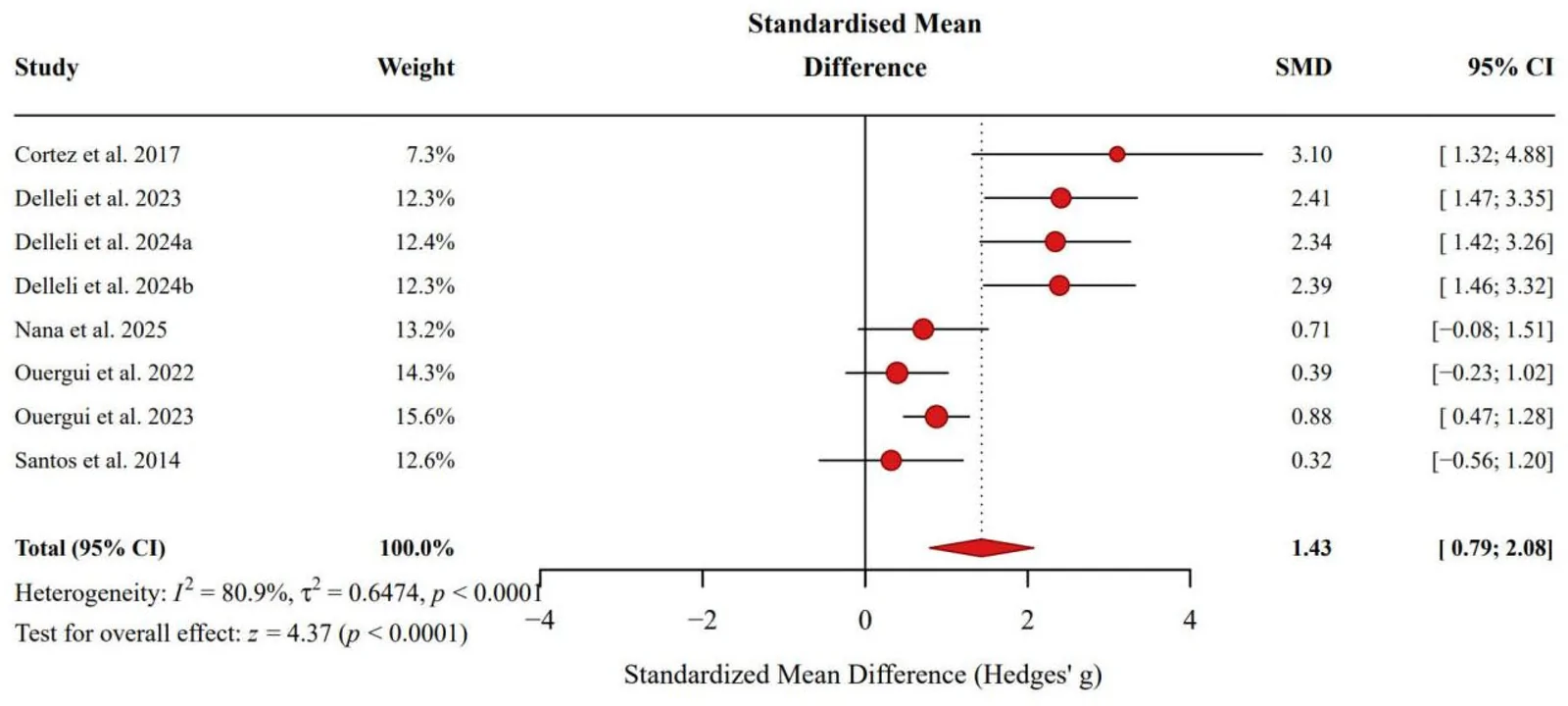
Forest plot of the effect of caffeine supplementation on reaction agility.

**Figure 3:**
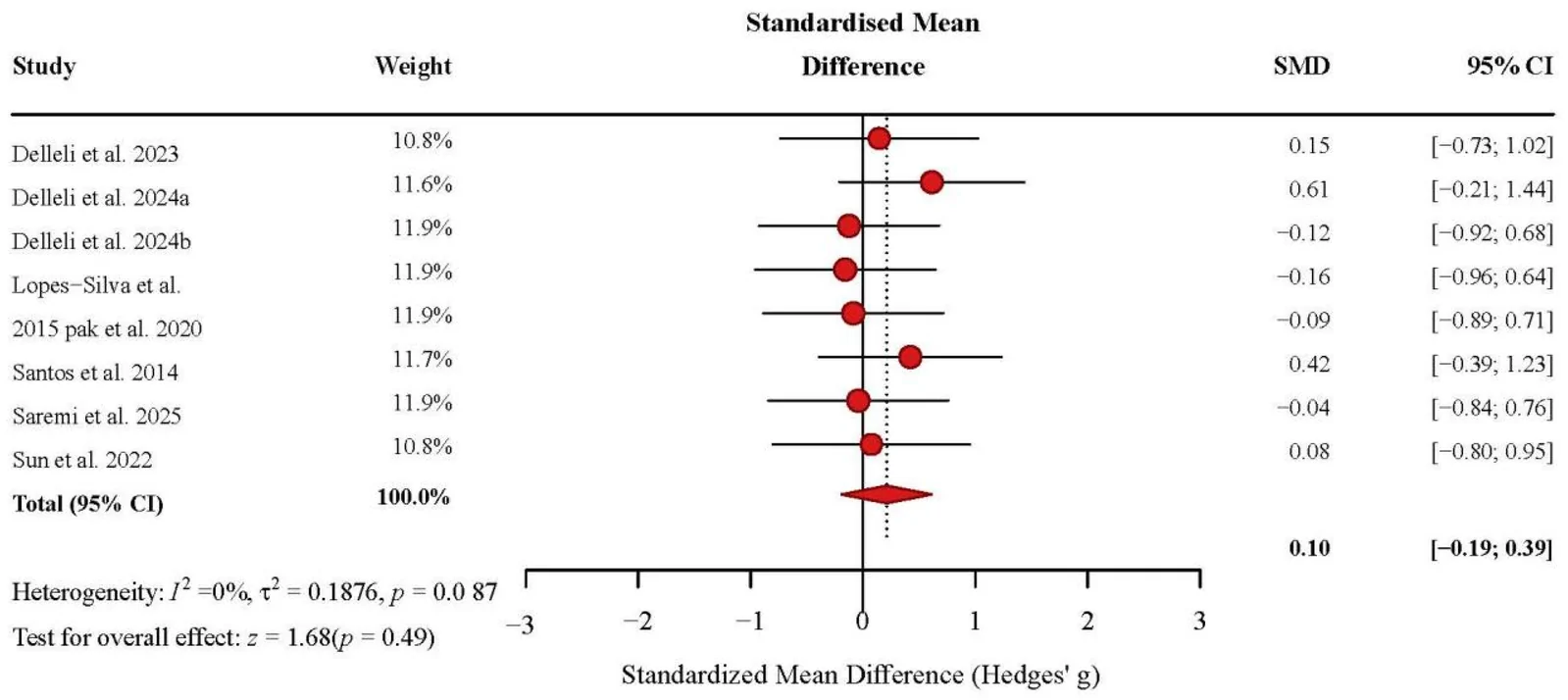
Forest plot of the effect of caffeine supplementation on endurance performance.

**Figure 4:**
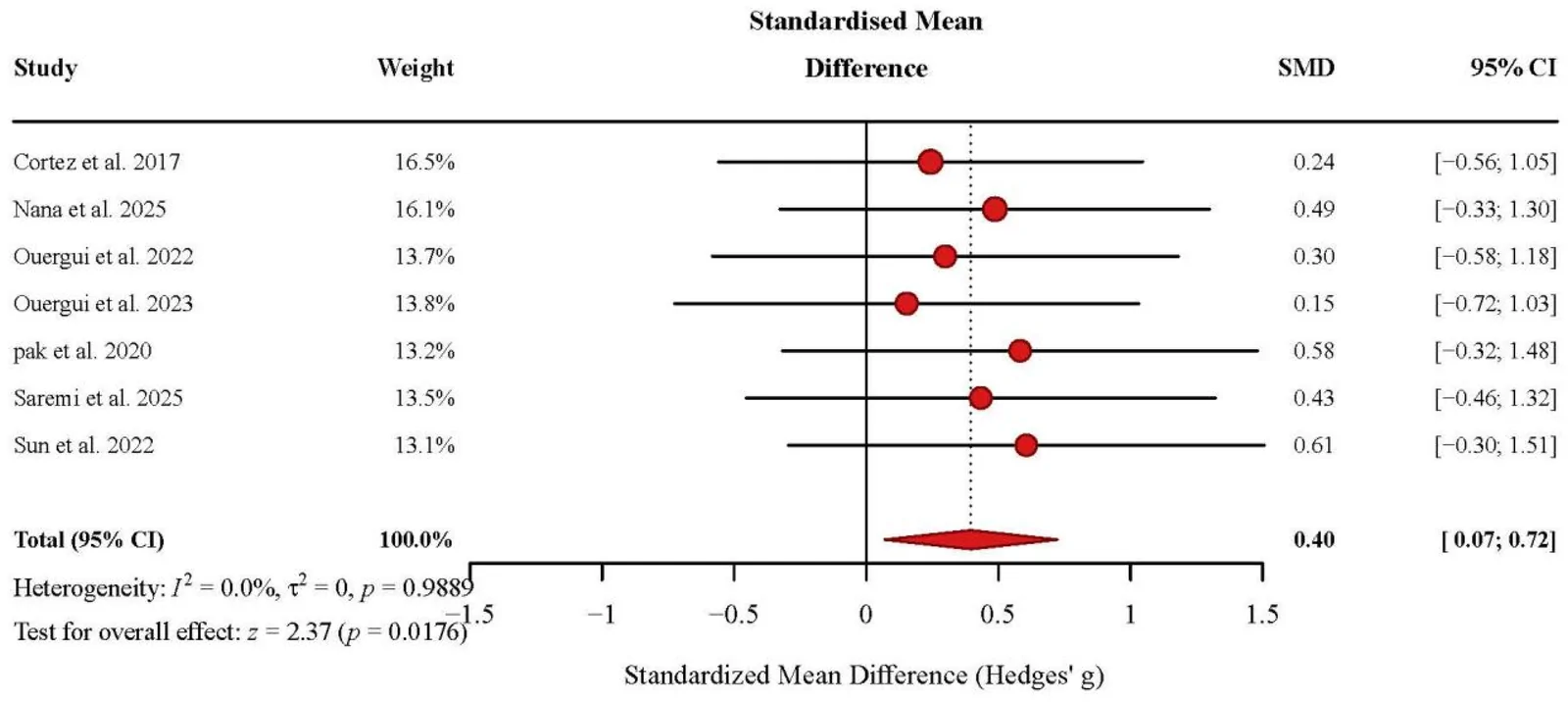
Forest plot of the effect of caffeine supplementation on explosive performance.

**Figure 5:**
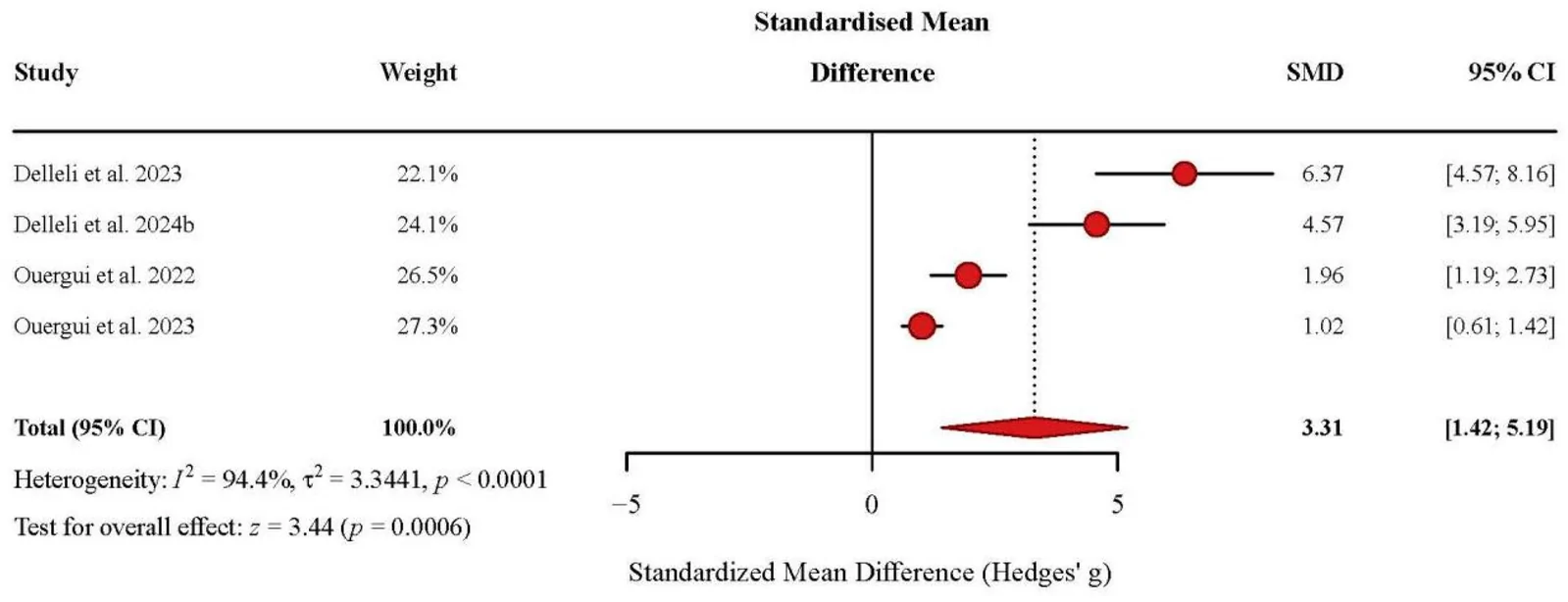
Forest plot of the effect of caffeine supplementation on FSKT-10s performance.

**Figure 6:**
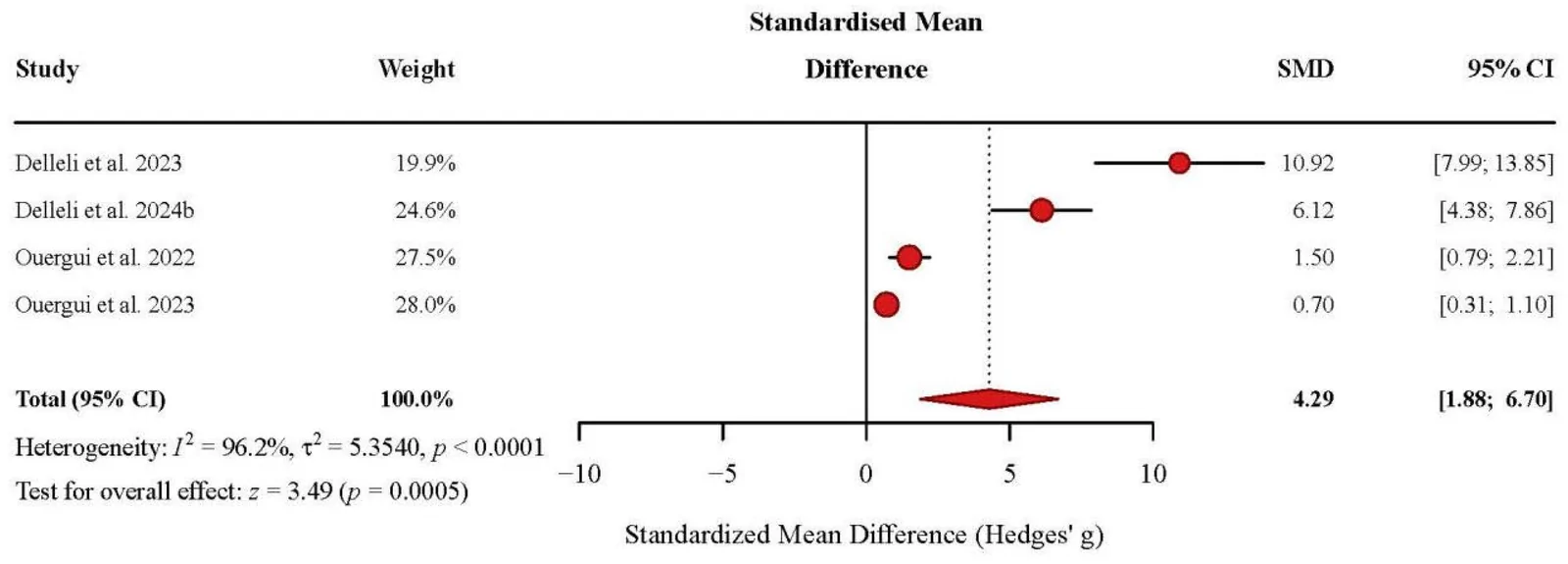
Forest plot of the effect of caffeine supplementation on FSKT-mult performance.

**Figure 7:**
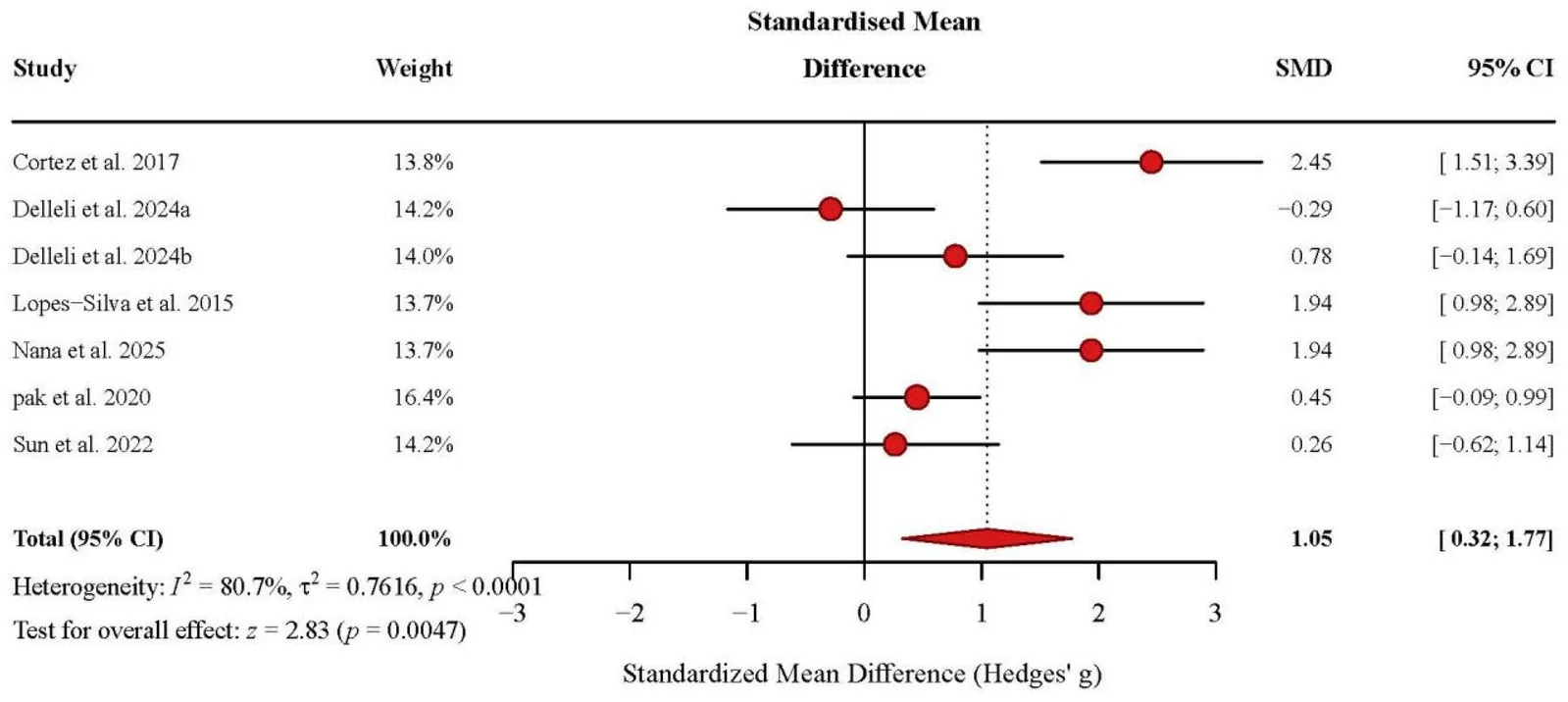
Forest plot of the effect of caffeine supplementation on technical accuracy.

The effects of caffeine intake on taekwondo athletes’ performance were synthesized across two broad domains: physical ability performance and technical performance, involving a total of six outcome indicators. The detailed pooled estimates are presented in Table 3, and the corresponding forest plots are shown in Figure 2, Figure 3, Figure 4, Figure 5, Figure 6 and Figure 7. In these forest plots, red circles indicate individual study effect sizes, with horizontal lines representing their 95% confidence intervals. Red diamonds represent the pooled effect estimates and their 95% confidence intervals. The vertical dotted lines indicate the pooled estimates, whereas the solid vertical lines at Hedges’ g = 0 represent no effect. Positive effect sizes indicate better performance following caffeine supplementation compared with placebo.

**Table 3:** Summary of the meta-analysis results.

Outcomes	No. of Studies (k)	*p* Value	I^2^	Model	Hedges’ g (95% CI)
physical ability					
Reaction Agility	8	<0.0001	81	RE	1.43 [0.79, 2.08]
Endurance	8	0.49	0	RE	0.10 [−0.19, 0.39]
Explosive	7	0.0176	0	RE	0.40 [0.07, 0.72]
Technical Performance					
FSKT-10s	4	0.0006	94	RE	3.31 [1.42, 5.19]
FSKT-mult	4	0.0005	96	RE	4.29 [1.88, 6.70]
Technical accuracy	7	0.0047	80	RE	1.05 [0.32, 1.77]

Note: Because heterogeneity was extremely high for FSKT-10s and FSKT-mult (I^2^ > 90%), these pooled estimates should be interpreted as exploratory average effects rather than as precise or stable common effects. The pooled estimates in Table 3 correspond to the main random-effects analysis presented in Figure 2, Figure 3, Figure 4, Figure 5, Figure 6 and Figure 7. Correlation-adjusted paired analyses assuming within-participant correlations of r = 0.30, 0.50, and 0.70 are reported separately as sensitivity analyses in Supplementary Table S2.

##### Effects of Caffeine on Taekwondo Physical Ability Performance

For reaction agility, eight studies were included, and caffeine supplementation was associated with a favorable pooled effect (Hedges’ g = 1.43, 95% CI 0.79 to 2.08; Figure 2). For endurance performance, eight studies were included, and no significant pooled effect was observed (Hedges’ g = 0.10, 95% CI −0.19 to 0.39; Figure 3). For explosive performance, seven studies were included, and caffeine supplementation was associated with improved performance (Hedges’ g = 0.40, 95% CI 0.07 to 0.72; Figure 4).

##### Effects of Caffeine on Taekwondo Technical Performance

For technical performance, the random-effects analysis presented in Table 3 showed favorable pooled effects for FSKT-10s (Hedges’ g = 3.31, 95% CI 1.42 to 5.19; Figure 5), FSKT-mult (Hedges’ g = 4.29, 95% CI 1.88 to 6.70; Figure 6), and technical accuracy (Hedges’ g = 1.05, 95% CI 0.32 to 1.77; Figure 7). Nevertheless, the FSKT estimates remained exceptionally large and heterogeneous and should therefore be interpreted as exploratory average effects rather than precise or stable common effects.

#### 3.4.2. Sensitivity Analyses

Sensitivity analyses were conducted using a leave-one-out approach for outcomes with three or more studies. After omitting each study in turn, the direction of the pooled effect generally remained unchanged, suggesting that the main findings were not driven by any single study. However, outcomes with substantial or very high heterogeneity, including reaction agility, technical accuracy, FSKT-10s, and FSKT-mult, showed greater fluctuation and should therefore be interpreted cautiously.

Test-type stratification was additionally considered for the heterogeneous reaction-agility and technical-accuracy domains. However, inspection of the study-level outcome assignments showed that most exact test types were represented by only one or two studies; therefore, separate pooled estimates would have been statistically unstable and were not calculated. The study-level test definitions and assignments are provided in Supplementary Table S4 to allow transparent interpretation of the domain-level estimates.

In the random-effects analysis summarized in Table 3 and Figure 2, Figure 3, Figure 4, Figure 5, Figure 6 and Figure 7, the pooled Hedges’ g values were 1.43 for reaction agility, 0.10 for endurance performance, 0.40 for explosive performance, 3.31 for FSKT-10s, 4.29 for FSKT-mult, and 1.05 for technical accuracy. Paired analyses assuming within-participant correlations of r = 0.30, 0.50, and 0.70 yielded the same direction of effect and statistical significance classification across all outcomes (Supplementary Table S2). These correlation-adjusted analyses were used to examine the sensitivity of pooled estimates to assumptions about within-participant dependence. However, this consistency should not be interpreted as evidence that the FSKT estimates were robust, because the effect magnitudes remained exceptionally large, heterogeneity remained very high, and the precise estimates and study weights depended partly on the assumed within-subject correlation. In an additional sensitivity analysis using REML estimation with Hartung–Knapp-adjusted confidence intervals, the pooled effect remained positive for FSKT-10s (Hedges’ g = 3.37, 95% CI −0.49 to 7.23) and FSKT-mult (Hedges’ g = 4.65, 95% CI −2.77 to 12.07). However, both confidence intervals crossed the null, indicating that statistical significance was not retained under this more conservative specification.

As a targeted influence analysis, the study with the largest study-level Hedges’ g was excluded from each FSKT meta-analysis. After exclusion, the pooled effect was attenuated but remained positive for FSKT-10s (Hedges’ g = 2.38, 95% CI 0.78 to 3.98, *p* = 0.0035; I^2^ = 92%) and FSKT-mult (Hedges’ g = 2.52, 95% CI 0.60 to 4.43, *p* = 0.0099; I^2^ = 95%). Thus, although the direction of effect was unchanged, the reduction in effect magnitude and the persistence of very high heterogeneity indicate that the large pooled FSKT estimates are not stable estimates of effect magnitude.

Cross-publication assessment did not identify any pair of studies for which participant reuse or duplicate cohort reporting could be established from the information reported in the original publications. Therefore, no individual study was excluded solely on the assumption of participant overlap. Because participant-level identifiers were unavailable for some reports, incomplete verification of non-overlap is acknowledged as a limitation rather than treated as evidence of duplicate sampling.

### 3.5. Publication Bias

**Figure 8:**
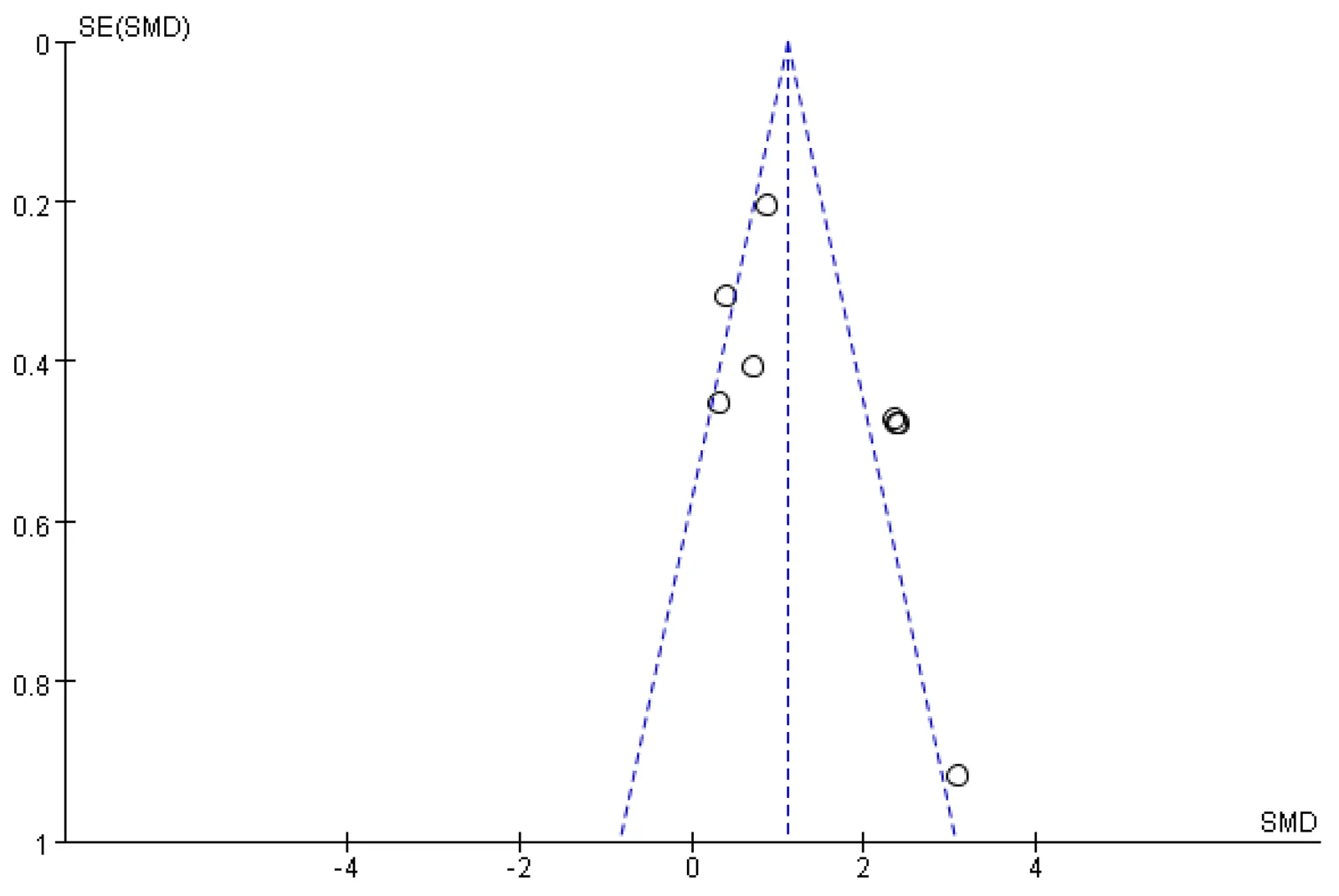
Funnel plot of the effect of caffeine supplementation on reaction agility. The vertical blue dashed line indicates the pooled effect estimate, while the two diagonal blue dashed lines represent the pseudo-95% confidence limits around the pooled estimate. Open circles represent individual studies.

**Figure 9:**
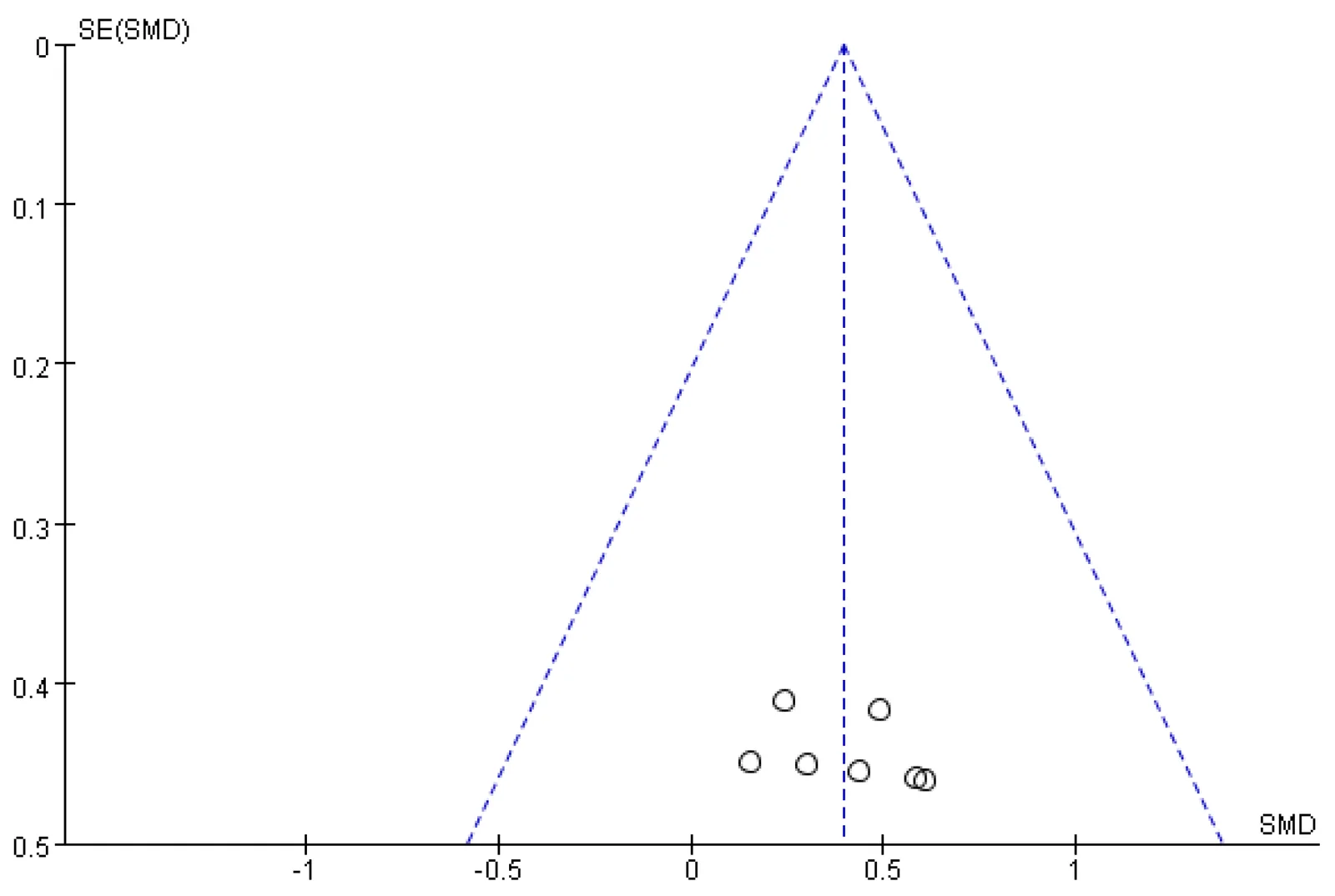
Funnel plot of the effect of caffeine supplementation on explosive performance. The vertical blue dashed line indicates the pooled effect estimate, while the two diagonal blue dashed lines represent the pseudo-95% confidence limits around the pooled estimate. Open circles represent individual studies.

**Figure 10:**
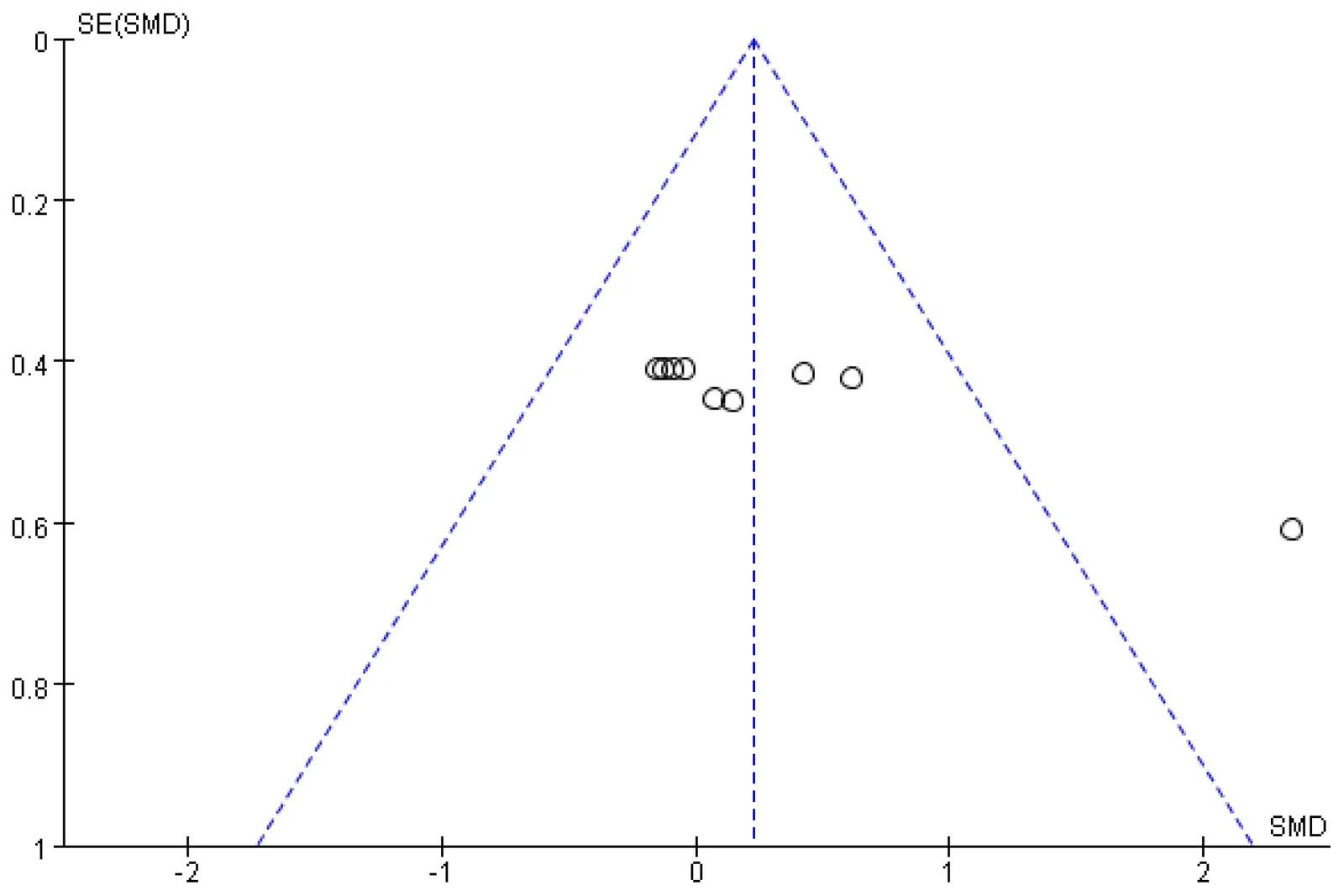
Funnel plot of the effect of caffeine supplementation on endurance performance. The vertical blue dashed line indicates the pooled effect estimate, while the two diagonal blue dashed lines represent the pseudo-95% confidence limits around the pooled estimate. Open circles represent individual studies.

**Figure 11:**
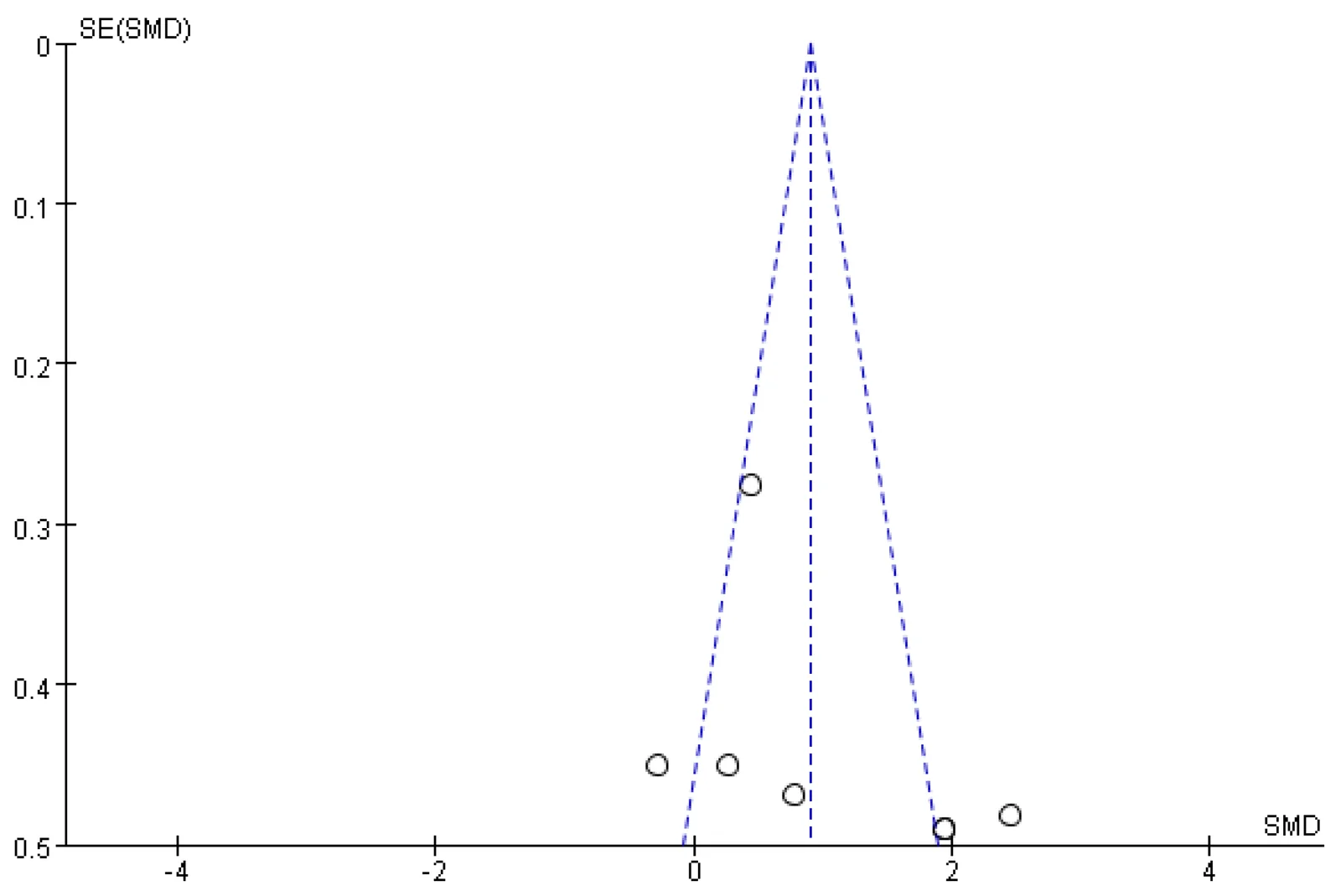
Funnel plot of the effect of caffeine supplementation on technical accuracy. The vertical blue dashed line indicates the pooled effect estimate, while the two diagonal blue dashed lines represent the pseudo-95% confidence limits around the pooled estimate. Open circles represent individual studies.

Because fewer than 10 studies contributed to each outcome, the funnel plots were considered exploratory descriptive displays only. Under these conditions, visual symmetry or asymmetry cannot be interpreted reliably. Therefore, no reliable conclusion regarding the presence or absence of publication bias could be drawn for any outcome. The funnel plots are presented in Figure 8, Figure 9, Figure 10 and Figure 11 without classifying publication-bias risk on the basis of their visual appearance.

### 3.6. Grade Recommendations

The strength of evidence according to GRADE criteria generated by the GRADEpro website (https://www.gradepro.org/ accessed on 10 April 2026) is listed in Table 4.

**Notes:** (a) **Inconsistency:** Rated as serious for reaction agility and technical accuracy due to substantial between-study heterogeneity, and very serious for FSKT-10s and FSKT-mult due to extremely high heterogeneity and unstable effect estimates. (b) **Indirectness:** Rated as serious because most outcomes were assessed using laboratory-based or simulated-performance tests that may not fully reflect the tactical, psychological, and physiological demands of official taekwondo competitions. (c) **Imprecision:** Rated as serious for endurance performance because the 95% confidence interval crossed the null effect, and for FSKT-10s and FSKT-mult because of the limited number of studies, relatively small sample sizes, and wide confidence intervals. (d) **Publication bias:** Could not be reliably assessed because fewer than 10 studies contributed to each outcome. No additional downgrade was applied in the absence of specific evidence of publication bias.

Reaction agility and technical accuracy were rated as low-certainty evidence because substantial between-study inconsistency was accompanied by indirectness when standardized or simulated performance tests were extrapolated to official competition. Explosive performance was rated as moderate-certainty evidence: the findings were statistically consistent, but the evidence was downgraded by one level for indirectness. Endurance performance was rated as low-certainty evidence because of indirectness and imprecision, as the confidence interval crossed the null and included both a small unfavorable effect and a potentially favorable effect.

FSKT-10s and FSKT-mult were rated as very-low-certainty evidence. The previous judgment of very serious inconsistency was retained, and the evidence was additionally limited by indirectness and imprecision arising from the small evidence base, wide confidence intervals, and uncertainty regarding the stability and real-world interpretation of the very large standardized effects. Although publication bias could not be reliably assessed for any outcome because fewer than 10 studies contributed to each meta-analysis, no additional downgrade was applied solely on this basis in the absence of specific evidence of selective non-publication.

Overall, no outcome was supported by high-certainty evidence. The revised GRADE assessment indicates possible favorable effects of caffeine supplementation on several taekwondo-specific performance measures, but the magnitude and generalizability of these effects remain uncertain, particularly for FSKT outcomes. The pooled estimates should therefore be interpreted as average effects under the studied experimental conditions rather than as precise estimates of performance improvement in official competition.

## 4. Discussion

### 4.1. Main Findings

This systematic review and meta-analysis found that caffeine supplementation was associated with favorable effects in several taekwondo-specific performance outcomes, particularly technical execution and reaction-based outcomes. However, the magnitude of these effects was uncertain for outcomes with substantial or very high heterogeneity. These findings are partially consistent with previous research in combat sports; however, the magnitude and consistency of effects vary substantially across performance domains [4,35,36].

### 4.2. Effects on Physical Ability Outcomes

Several biologically plausible mechanisms may explain the observed associations, including adenosine-receptor antagonism, increased central nervous system excitability, reduced perceived exertion, and improved neuromuscular activation. However, these mechanisms were not directly tested in the included studies or in the present meta-analysis. Therefore, they should be regarded as possible explanations rather than findings of this review [4,38,40,41,42].

### 4.3. Interpreting Heterogeneity and Potential Mechanisms

Expectancy effects may also contribute to caffeine-related performance changes, particularly in outcomes requiring maximal voluntary effort. However, expectancy effects were not directly examined in the present meta-analysis, and their role in the included taekwondo studies remains speculative [19,35,38]. Systematic review evidence indicates that expectations of caffeine intake alone may improve exercise performance through reduced pain perception and enhanced attention [44]. Caffeine ingestion 70 min before exercise significantly reduces ratings of perceived exertion during steady-state exercise, which represents an important mechanism for improving subsequent high-intensity performance [45]. Nevertheless, the substantial heterogeneity observed suggests that individual characteristics and testing protocols may strongly influence the magnitude of these effects.

As a post hoc exploratory descriptive comparison, several low-to-moderate-dose protocols appeared to be associated with favorable outcomes in the included studies. However, no formal dose–response meta-analysis or subgroup interaction test was performed. Caffeine dose was strongly confounded with age, research team or study site, participant characteristics, training status, co-interventions, and outcome type; therefore, the observed between-study pattern cannot be attributed to caffeine dose independently. These observations should be regarded only as hypothesis-generating signals for future studies rather than evidence of an optimal or superior caffeine dose [25,41]. Habitual caffeine use alters physiological and cognitive responses to acute caffeine intake, with this habituation potentially mediated by mechanisms such as adenosine receptor upregulation and enhanced cytochrome P450 enzyme activity [46]. External literature provides a broader context for possible dose-dependent responses, but these findings should not be used to infer a dose–response relationship within the present taekwondo dataset. Systematic reviews of endurance sports have also found that caffeine exhibits stable ergogenic effects within moderate dose ranges with good reproducibility [47]. Relatively lower doses can promote focus and reaction speed but have limited effects on strength or endurance enhancement [40]. At higher intake levels, individual differences become pronounced, with some athletes experiencing anxiety, increased heart rate, or impaired coordination [38,39,48]. Research indicates that caffeine may produce side effects including tachycardia, palpitations, nervousness, gastrointestinal discomfort, headaches, and sleep disturbances within ergogenic dose ranges, with incidence and severity showing dose-dependent patterns—adverse event rates of 6–34% at ≤3.0 mg/kg versus 8–83% at ≥6.1 mg/kg [24]. External literature has proposed possible dose-related patterns; however, the present review did not formally evaluate an optimal dose [49]. Although these physiological mechanisms may theoretically support endurance performance, the present meta-analysis did not demonstrate a statistically significant improvement in endurance-related outcomes in taekwondo athletes. Therefore, caffeine’s effects on endurance performance in this sport remain uncertain.

Interpretation of apparent age- or dose-related patterns is complicated by the concentration of several studies within closely affiliated research teams. A substantial proportion of studies involving younger athletes used 3 mg/kg caffeine and similar taekwondo-specific testing protocols [37,38,39,40,43]; however, this pattern was not universal across the evidence base, as other studies involving younger athletes used different caffeine protocols [36]. Consequently, age, caffeine dose, research team or study site, participant characteristics, and testing protocol were strongly correlated across studies rather than independently distributed. The available data therefore do not permit reliable separation of their individual contributions to between-study differences in effect magnitude.

Importantly, concentration of evidence within affiliated research teams does not itself demonstrate participant overlap or duplicate publication. Our cross-publication assessment of the closely affiliated reports [37,38,39,40,43] identified no direct evidence that the same participant cohort was repeatedly analyzed. Nevertheless, incomplete reporting of recruitment periods and the absence of participant-level identifiers in some reports limited definitive cross-study verification. Irrespective of participant overlap, the concentration of evidence within similar populations, research settings, and experimental protocols reduces the degree of independent replication and therefore limits external generalizability. Apparent age- or dose-related differences should consequently be considered descriptive and hypothesis-generating rather than evidence of independent effect modification.

### 4.4. Effects on Technical Performance Outcomes

Although comparisons with other combat sports help contextualize the findings, the present discussion primarily focuses on interpreting caffeine’s effects within the unique technical and physiological demands of taekwondo. In addition, the high heterogeneity observed in several key outcomes indicates that pooled estimates may be strongly influenced by between-study differences in methods, participants, and protocols, which limits the generalizability of the findings.

The findings of this study are highly consistent with previous systematic reviews in combat sports. Diaz-Lara’s meta-analysis indicated that caffeine significantly enhances strength, power, and reactive ability across various combat sports [48]. A relevant network meta-analysis found that caffeine and its combination with other supplements (such as sodium bicarbonate) demonstrated the greatest potential for improving comprehensive performance [49]. Caffeine may influence power-related outcomes in some combat sports contexts, while its more consistent effects in taekwondo appear to relate to tactical execution and rhythm control [39]. These findings collectively indicate that caffeine’s ergogenic mechanisms exhibit cross-sport consistency, but taekwondo’s high tempo, short-duration explosive efforts, and technical complexity make caffeine’s central activation effects particularly prominent. The extremely large effect sizes observed for FSKT-based outcomes should be interpreted cautiously, given the limited number of studies, very high heterogeneity, and the concentration of evidence from a small number of research groups.

From a practical perspective, caffeine may represent a potentially useful nutritional ergogenic strategy, though the magnitude of benefit in competition settings is likely more modest than the laboratory-derived effect sizes suggest [50]. Given significant individual variability, athletes should avoid “one-size-fits-all” protocols and determine optimal strategies through personalized testing [46]. Multiple studies demonstrate that appropriate pre-competition caffeine intake can significantly improve athletes’ power output, reaction speed, and technical performance without increasing burden [22]. In the included studies, caffeine was administered 45–70 min before exercise. However, because the number of studies was limited and administration timing was not systematically varied, no formal timing-effect analysis was conducted. A study reported that dosing 60–70 min before exercise can optimize performance, but this finding needs to be independently verified [45]. For individuals with lower tolerance or anxiety sensitivity, appropriately reduced intake can still yield positive psychological and neural effects. Given that the risk of adverse events with high-dose caffeine is markedly higher than with low doses, athletes should fully consider individual tolerance and potential adverse reaction risks while pursuing performance enhancement [24]. Additionally, research shows that combining multiple strategies such as music stimulation, sodium bicarbonate, or short-duration high-intensity warm-up can further amplify caffeine’s neural activation and psychological arousal effects [35,49]. Notably, short-term (less than one week) pre-competition caffeine withdrawal strategies do not restore or enhance caffeine’s ergogenic effects; rather, they may negatively impact athletes’ subjective state and performance confidence due to withdrawal symptoms (such as headaches, fatigue, irritability) [47]. Post hoc exploratory descriptive comparisons suggested that favorable outcomes occurred in several studies using 3–5 mg/kg caffeine and that some studies involving younger or more highly trained athletes reported relatively large effects. However, these observations were not derived from prespecified subgroup analyses, meta-regression, or formal tests of interaction. Moreover, caffeine dose, age, training status, research team or study site, and testing protocol were strongly confounded across studies. Consequently, the observed between-study differences cannot be interpreted as independent dose-, age-, or training-status effects. They should be considered hypothesis-generating only and require confirmation in adequately powered studies that vary these factors independently.

#### Interpretation of Large Effect Sizes in FSKT Outcomes

The exceptionally large standardized effects observed for FSKT-10s and FSKT-mult require cautious methodological interpretation. Hedges’ g expresses the between-condition mean difference relative to the pooled condition-specific standard deviation; therefore, when within-condition variability is small, even a modest absolute difference in kick counts can yield a very large standardized effect [50]. This issue is particularly relevant to FSKT outcomes, which are highly standardized maximal-frequency kicking tasks. Inspection of the contributing studies showed relatively narrow condition-specific variability in several FSKT datasets; for example, standard deviations of approximately 1–2 kicks were reported for FSKT-10s in some caffeine and placebo conditions [34,35]. Consequently, the very large Hedges’ g may partly reflect the combination of a genuine between-condition difference and a small standardizing denominator, and should not be interpreted as proportional real-world improvements in kicking performance [46,47].

Crossover correlation represents a separate source of analytical uncertainty. Within-participant dependence was examined in paired sensitivity analyses assuming correlations of r = 0.30, 0.50, and 0.70, rather than being incorporated into the variance estimates underlying the main forest plots. Under the effect-size formulation used in this review, the assumed correlation primarily affects the variance, standard error, confidence interval, and study weight rather than directly changing the condition-specific standard deviation used to standardize the mean difference. Accordingly, failure to account for within-participant correlation can shift the pooled estimate indirectly through changes in precision and weighting, but is unlikely to be the principal source of the exceptionally large study-level FSKT Hedges’ g [27, 30]. Consistent with this interpretation, the pooled estimates remained large across the examined correlation assumptions (FSKT-10s: Hedges’ g = 2.58–3.79; FSKT-mult: Hedges’ g = 3.07–4.35), although their exact magnitude remained assumption-dependent.

The targeted exclusion of the highest-effect study attenuated both pooled FSKT estimates but did not materially resolve heterogeneity. Taken together with the loss of statistical significance under the REML–Hartung–Knapp specification, these findings indicate that the quantitative synthesis is most appropriately interpreted as an exploratory summary of effect direction and approximate magnitude rather than as evidence of a stable common effect.

Two reviewers rechecked the extracted means, standard deviations, sample sizes, outcome definitions, and direction coding against the original reports. FSKT-10s kick counts were consistently coded so that higher values represented better performance, whereas decrement indices in FSKT-mult were direction-reversed where required. We also compared the available recruitment and participant information across publications and found no explicit evidence of overlapping samples. The direction of the pooled effects was unchanged across the examined within-subject correlation assumptions. However, the additional REML random-effects sensitivity analysis with Hartung–Knapp-adjusted confidence intervals yielded positive but non-significant estimates for FSKT-10s (Hedges’ g = 3.37, 95% CI −0.49 to 7.23) and FSKT-mult (Hedges’ g = 4.65, 95% CI −2.77 to 12.07). Thus, although the point estimates remained large, their statistical significance was not retained under this more conservative specification, reinforcing the need for cautious interpretation.

All four FSKT studies originated from a small number of research teams using broadly similar populations and protocols. Shared research teams do not by themselves establish statistical dependence, but the concentration of evidence limits independent replication and external generalizability. In addition, the ecological validity of these outcomes should be considered. Although some included studies incorporated simulated-contest or combat-related measures [1,23,34], most performance outcomes were assessed using standardized laboratory-based or simulated tasks rather than official competition. Such tests can isolate specific attributes such as reaction speed, kicking frequency, power, or technical execution, but they do not fully reproduce the opponent-dependent tactical decisions, unpredictable attack–counterattack exchanges, psychological stress, scoring contingencies, and cumulative fatigue encountered during actual taekwondo competition. Importantly, even taekwondo-specific exercise protocols with activity patterns similar to competition have been shown to elicit lower cardiovascular, metabolic, and catecholamine responses than championship combat [50]. Furthermore, successive combats performed within a competition-like schedule produce changing physiological and hormonal responses across bouts and recovery periods [51]. Therefore, improvements observed in standardized reaction, kicking-frequency, or other laboratory-based outcomes should not be assumed to translate proportionally into competitive performance. The pooled Hedges’ g should be interpreted as effects under controlled testing conditions, and the magnitude of any benefit in real competition may be attenuated, modified, or potentially offset by tactical, psychological, opponent-related, and accumulated-fatigue demands.

### 4.5. Safety and Adverse Events

This review did not conduct a systematic meta-analysis of adverse events but provided a descriptive summary of safety reporting in included studies. Of 12 studies, 7 (58.3%) explicitly reported no observed adverse events, 3 (25.0%) reported mild symptoms primarily including gastrointestinal discomfort (diarrhea), headaches, and tachycardia, and 2 (16.7%) did not report safety data. Although the studies reporting adverse events used 3 mg/kg doses, this should not be interpreted as evidence that higher doses are safer, because adverse-event monitoring and reporting were inconsistent across studies. Due to significant heterogeneity in adverse event monitoring and reporting methods across original studies (active monitoring vs. passive reporting, standardized assessment vs. descriptive recording), accurate estimation of adverse event incidence or severity was not possible. Importantly, existing literature indicates that even within routine ergogenic dose ranges (3–9 mg/kg), caffeine may cause various side effects, including cardiovascular responses (tachycardia, palpitations), gastrointestinal symptoms, neurological symptoms (nervousness, anxiety), headaches, and sleep disturbances, with dose-dependent patterns showing significantly higher adverse event rates at high doses (≥6.1 mg/kg, 8–83%) compared to low doses (≤3.0 mg/kg, 6–34%) [25]. Therefore, athletes and coaches should carefully weigh potential benefits against risks when considering caffeine supplementation, start with conservative doses, monitor individual responses, and be aware of individual tolerance variability [52].

### 4.6. Limitations and Future Directions

This study has several limitations. (1) Sample representativeness was limited: sample sizes were small, gender distribution was uneven, and a substantial proportion of the evidence originated from closely affiliated research teams using similar populations and experimental protocols. Cross-publication assessment identified no direct evidence of participant overlap or duplicate cohort reporting. However, participant-level identifiers and complete recruitment-period information were unavailable for some reports, limiting definitive verification across publications. More importantly, the concentration of evidence within similar research settings reduces independent replication and limits external generalizability. (2) Age, caffeine dose, research team or study site, sex, training status, and testing protocol were strongly correlated across studies and could not be reliably evaluated as independent effect modifiers. Therefore, observed between-study differences cannot be attributed specifically to age or caffeine dose, and any such descriptive patterns should be considered hypothesis-generating. (3) Most studies focused on short-term interventions, limiting insight into long-term training adaptations. (4) For crossover trials, the main analysis used condition-level variance calculations without explicitly accounting for within-participant correlation. Because the original reports generally did not provide paired-difference standard deviations or within-participant correlations, paired sensitivity analyses were performed using assumed correlations of r = 0.30, 0.50, and 0.70. Although the qualitative interpretation remained unchanged, uncertainty regarding the true within-participant correlations may affect sampling variances, study weights, and pooled effect estimates. This limitation should be considered when interpreting the precision and magnitude of the findings.

An additional limitation concerns the methodological quality of the available safety evidence.

Adverse-event monitoring was not standardized across the included studies, and the timing, frequency, ascertainment methods, and reporting thresholds were inconsistently described. Consequently, studies reporting no observed adverse events may not have captured all transient, delayed, or participant-unreported symptoms, and the incidence of adverse reactions may therefore have been underestimated. In addition, most available safety observations were derived from acute or relatively short-term supplementation protocols. The current evidence is therefore insufficient to comprehensively assess the safety risks associated with repeated or longer-term caffeine supplementation in taekwondo athletes. Future trials should incorporate predefined adverse-event criteria, standardized active monitoring at prespecified time points, and sufficient follow-up to evaluate cumulative or delayed adverse effects.

Ecological validity represents an additional limitation because most outcomes were derived from standardized laboratory or simulated-performance tests. Even when taekwondo-specific simulations were used, these settings could not fully reproduce the tactical uncertainty, competitive stress, opponent interaction, scoring consequences, and cumulative fatigue of official competition; therefore, laboratory-derived effect sizes should not be interpreted as proportional improvements in competition performance [50,51].

The use of broad functional outcome domains may have introduced conceptual heterogeneity because measures within a domain were related but not identical. For example, reaction-related outcomes included both response-time measures and sport-specific agility tests, whereas technical-accuracy outcomes included success-rate measures and broader simulated-competition performance indicators; these tests differ in their task demands and likely cognitive and neuromuscular contributions. Consequently, the pooled estimates should be interpreted at the broader domain level rather than as evidence for a specific test. Although narrower analyses according to the exact measurement instrument were considered, most resulting categories contained only one or two studies, precluding informative quantitative synthesis [36,37].

Future research should use larger, preregistered, and standardized trials to examine long-term adaptation, individual differences in caffeine response, habitual caffeine intake, adverse-event monitoring, and potential genetic moderators such as CYP1A2 and ADORA2A [46].

The review was restricted to English-language publications because of the language resources available for full-text assessment and data extraction. This restriction may nevertheless have introduced language bias and resulted in the omission of relevant studies published in other languages.

Overall, the available evidence suggests that caffeine supplementation, across the acute and longer-term or repeated intervention protocols included in this review, is associated with improvements in selected reaction-based and technical performance outcomes in taekwondo athletes. Its ergogenic mechanisms arise from multidimensional effects including enhanced muscle activation, increased central excitability, and reduced subjective fatigue [39]. Simultaneously, the non-negligible psychological component of caffeine effects suggests that athletes’ expectations, beliefs, and prior experiences play important roles in performance enhancement, a finding that provides new insights for developing comprehensive nutrition-psychology intervention strategies [44].

## 5. Conclusions

Across the mixed acute and longer-term or repeated protocols included in this review, caffeine supplementation was associated with favorable pooled effects for some taekwondo-specific performance outcomes, particularly explosive and reaction-related measures. No significant pooled effect was observed for endurance performance. However, these estimates represent averages across clinically heterogeneous interventions and should not be interpreted as evidence of a single uniform caffeine effect.

For technical performance outcomes, including technical accuracy, FSKT-10s, and FSKT-mult, the pooled results suggested favorable effects; however, these findings should be interpreted with caution. The magnitude and generalizability of these effects remain uncertain because of substantial or very high heterogeneity, the small number and concentration of contributing studies, the handling of crossover trials, and variation in intervention protocols, populations, doses, co-interventions, and outcome tests.

Descriptive patterns regarding dose, age, training status, or protocol characteristics should be considered post hoc and hypothesis-generating only, because formal subgroup, interaction, or dose–response analyses were not conducted and these study characteristics were strongly confounded. At present, caffeine supplementation may have potential for improving selected taekwondo performance measures; however, the extent to which laboratory- or simulation-based improvements translate into official competition performance remains uncertain. Further independent, standardized, and adequately powered trials are needed to clarify the effects of caffeine supplementation across different taekwondo populations and testing conditions.

## Figures and Tables

**Table 1:** PICO framework for evaluating the effects of caffeine supplementation on performance in taekwondo athletes.

PICO Component	Final Definition in This Review
**P (Population)**	Taekwondo athletes of all competitive levels (amateur/professional/elite), with no restriction on age or sex.
**I (Intervention)**	Caffeine administered alone or in combination with another modality, provided that the co-intervention was identical in the matched placebo condition.
**C (Comparison)**	A matched placebo condition in which training, testing procedures, and any co-interventions were identical to the caffeine condition, with caffeine replaced by placebo.
**O (Outcomes)**	Quantitative taekwondo performance outcomes with sufficient data for meta-analysis, synthesized into two overarching domains: **Physical ability** (Reaction Agility, Endurance, Explosive performance) and **Technical performance** (FSKT-10s, FSKT-mult, Technical accuracy).

**Table 4:** GRADE assessment of the certainty of evidence.

Outcome/Unique Participants (Studies)	Risk of Bias	Inconsistency	Indirectness	Imprecision	Publication Bias	Effect Estimate, Hedges’ g (95% CI)	Certainty (GRADE)
Reaction agility 156 (8 studies)	Not serious	Serious (a)	Serious (b)	Not serious	Unable to assess reliably (d); no downgrade	1.43 (0.79–2.08)	Low
Explosive performance 147 (7 studies)	Not serious	Not serious	Serious (b)	Not serious	Unable to assess reliably (d); no downgrade	0.40 (0.07–0.72)	Moderate
Endurance performance 117 (8 studies)	Not serious	Not serious	Serious (b)	Serious (c)	Unable to assess reliably (d); no downgrade	0.10 (−0.19–0.39)	Low
FSKT-10s 104 (4 studies)	Not serious	Very serious (a)	Serious (b)	Serious (c)	Unable to assess reliably (d); no downgrade	3.31 (1.42–5.19)	Very low
FSKT-mult 104 (4 studies)	Not serious	Very serious (a)	Serious (b)	Serious (c)	Unable to assess reliably (d); no downgrade	4.29 (1.88–6.70)	Very low
Technical accuracy 105 (7 studies)	Not serious	Serious (a)	Serious (b)	Not serious	Unable to assess reliably (d); no downgrade	1.05 (0.32–1.77)	Low

GRADE certainty (starting from RCTs = High): ⊕⊕⊕⊕ High; ⊕⊕⊕◯ Moderate; ⊕⊕◯◯ Low; ⊕◯◯◯ Very low. Note: Participant numbers refer to unique randomized participants contributing to each outcome. Participants in crossover trials were counted once rather than once per intervention condition. The same participant may contribute to more than one outcome row; therefore, participant numbers should not be summed across outcomes.

## Data Availability

All data generated or analyzed during this study are included in this published article and its supplementary information files. The datasets extracted from the included studies are available from the corresponding author upon reasonable request.

## Supplementary Materials

The following supporting information can be downloaded at: https://www.mdpi.com/article/10.3390/metabo16100784/s1, Table S1. The search strategy used for each database; Table S2. Crossover sensitivity analysis summary; Table S3. PEDro; Table S4. Operational definitions and outcome assignment for each meta-analysis.

## Abbreviations

The following abbreviations are used in this manuscript:

Abbreviations	
CI	Confidence interval
FSKT-10s	10 s Frequency Speed of Kick Test
FSKT-mult	Multiple Frequency Speed of Kick Test
GRADE	Grading of Recommendations Assessment, Development and Evaluation
PAPE	Post-activation performance enhancement
PEDro	Physiotherapy Evidence Database
PICO	Population, Intervention, Comparison, Outcomes
PRISMA	Preferred Reporting Items for Systematic Reviews and Meta-Analyses
RCT	Randomized controlled trial
RE	Random-effects model
SMD	Standardized mean difference
TSAT	Taekwondo-Specific Agility Test
VO_2_max	Maximal oxygen uptake
VT2	Second ventilatory threshold
mg/kg	Milligrams per kilogram of body mass
W	Watt
W/kg	Watts per kilogram
s	Second
↑	Statistically significant increase or improvement
→	No statistically significant difference
↓	Statistically significant decrease
