# Supplementary material for: Caffeine Intake and Performance Outcomes in Taekwondo Athletes: A Systematic Review and Meta-Analysis
Source: Metabolites. 2026 Oct 16;16(10):784. doi: 10.3390/metabo16100784 (PMC13707372; doi:10.3390/metabo16100784)
Supplement: Supplementary file 1 [file metabolites-16-00784-s001.zip › metabolites-4610229-supplementary.pdf]

Caffeine intake and performance outcomes in taekwondo athletes: a systematic review and meta-analysis

Limin Cai[1,†], Jintao Guo[2,†], Si Chen[3*]

1. North China Elect Power Univ, Dept Phys Educ, Beijing, Peoples R China.
2. Shandong Yingcai University, Jinan, China
3. Zhejiang Normal Univ, Coll Phys Educ & Hlth Sci, Jinhua, Zhejiang, Peoples R China.

† These authors contributed equally to this work and share first authorship.

*Correspondence:

Si Chen, College of Physical Education and Health Sciences, Zhejiang Normal University, Jinhua, China.

Email: cstkd@zjnu.edu.cn

Table S1. The search strategy used for each database

Databases.	Keywords	Search date	Results
Pubmed	(("combat sport*" OR "martial art*" OR taekwondo OR "tae kwon do" OR karate OR judo OR boxing OR kickboxing OR "mixed martial art*" OR MMA OR wrestling) AND (caffeine OR coffee OR methylxanthine* OR "energy drink*") AND (performance OR athlete* OR exercise OR sport*))	April 2026	62
Web of science	TS=(taekwondo OR "tae kwon do" OR TKD) AND TS=(caffeine OR coffee OR methylxanthine*) AND TS=(athlete* OR performance OR sport*)<br>(taekwondo OR "tae kwon do" OR TKD) AND (caffeine OR "caffeine supplementation" OR "caffeine ingestion")	April 2026	47

SPORTDiscus (EBSCO)	(caffeine OR "caffeine supplementation" OR "caffeine intake" OR "caffeine ingestion") AND (taekwondo OR "tae kwon do" OR "martial arts" OR "combat sports" OR "fighting sports") AND (performance OR "athletic performance" OR power OR strength OR speed OR agility OR endurance OR "reaction time" OR ergogenic)	April 2026	83
Cochrane Library	#1 (caffeine OR coffee OR "caffeine supplementation" OR "caffeine intake" OR "caffeine ingestion"):ti,ab,kw #2 (taekwondo OR "combat sport*" OR "martial art*" OR karate OR judo OR "mixed martial arts" OR "kick boxing" OR boxing OR wrestling OR "Brazilian jiu jitsu"):ti,ab,kw #3 (performance OR "exercise performance" OR "sports performance" OR "physical performance" OR "reaction time" OR agility OR speed OR strength OR power OR endurance OR "time to exhaustion" OR "anaerobic capacity" OR "aerobic capacity"):ti,ab,kw #4 #1 AND #2 AND #3	April 2026	47
	Supplementary sensitivity search: (taekwondo OR taekwon-do OR combat sport OR FSKT OR "Frequency Speed of Kick Test") AND (caffeine OR coffee OR methylxanthine OR supplementation OR ergogenic aid). No additional eligible studies were identified.		

Table S2. Crossover sensitivity analysis summary

Outcome	Primary analysis SMD	r = 0.3	Primary paired analysis r = 0.5	r = 0.7	Interpretation
Reaction agility	1.43 [0.79, 2.07]	1.16 [0.64, 1.67]	1.33 [0.77, 1.90]	1.64 [0.99, 2.30]	Direction unchanged; remains significant
Endurance performance	0.10 [−0.19, 0.39]	0.08 [−0.13, 0.28]	0.09 [−0.12, 0.30]	0.11 [−0.12, 0.34]	Direction unchanged; remains non-significant

Explosive performance	0.40 [0.07, 0.72]	0.31 [0.08, 0.55]	0.36 [0.13, 0.60]	0.45 [0.21, 0.69]	Direction unchanged; remains significant
FSKT-10s	3.30 [1.42, 5.19]	2.58 [1.17, 4.00]	3.01 [1.40, 4.63]	3.79 [1.81, 5.77]	Direction unchanged; remains significant, but very large and heterogeneous
FSKT-mult	4.28 [1.88, 6.69]	3.07 [1.39, 4.75]	3.52 [1.65, 5.40]	4.35 [2.11, 6.59]	Direction unchanged; remains significant, but very large and heterogeneous
Technical accuracy	1.05 [0.32, 1.77]	0.80 [0.25, 1.34]	0.90 [0.30, 1.51]	1.08 [0.38, 1.78]	Direction unchanged; remains significant

Table S3. PEDro

**Study**	**Eligibility criteria**	**Random allocation**	**Concealed allocation**	**Similar baseline**	**Participant blinding**	**Investigator blinding**	**Assessor blinding**	**Completeness of follow-up**	**Intention to treat**	**Between group comparisons**	**Point measures and variability**	**Total Score**	**Overall quality**
Cortez et al . 2017	1	1	1	0	1	1	0	1	0	1	1	7	High quality (low risk of bias)
Delleli et al. 2023	1	1	1	0	1	1	0	1	0	1	1	7	High quality (low

													risk of bias)
Delleli et al. 2024a	1	1	1	0	1	1	1	1	0	1	1	8	High qualit y (low risk of bias)
Delleli et al. 2024b	1	1	1	1	1	1	0	1	1	1	1	9	High qualit y (low risk of bias)
LopesSil va et al. 2015	1	1	1	1	0	1	0	1	1	1	1	8	High qualit y (low risk of bias)
Nana et al. 2025	1	1	1	1	1	1	0	1	1	1	1	9	High qualit y (low risk of bias)

Ouergui et al. 2022	1	1	1	1	1	1	0	1	1	1	1	9	High quality (low risk of bias)
Ouergui et al. 2023	1	1	1	1	1	1	0	1	1	1	1	9	High quality (low risk of bias)
pak et al. 2020	1	1	1	1	1	0	0	1	1	1	1	8	High quality (low risk of bias)
Santos et al. 2014	1	1	1	1	1	1	1	1	0	1	1	9	High quality (low risk of bias)

Saremi et al. 2025	1	1	1	1	1	1	1	1	0	1	1	9	High qualit y (low risk of bias)
Sun et al., 2022	1	1	1	1	1	1	1	0	0	1	1	8	High qualit y (low risk of bias)

Table S4. Operational definitions and outcome assignment for each meta-analysis

**Study**	**Meta-analysis domain**	**Exact outcome used**	**Unit**	**Direction indicating improvement**	**Handling of multiple outcomes**
Santos et al. 2014	Reaction agility	Reaction time / reaction agility	s or ms	Lower value = better; effect direction reversed	Selected
Santos et al. 2014	Technical accuracy	Kick time / simulated-contest technical indicator, if used	s or score	Lower time or higher score = better	Selected or combined
Lopes-Silva et al. 2015	Endurance performance	Time/fatigue-related combat-simulation indicator	s or physiological unit	Higher endurance value or lower fatigue value = better	Selected
Sun et al. 2022	Explosive performance	Peak power / peak power relative to body mass	W or W/kg	Higher value = better	Combined if multiple power

Study	Meta-analysis domain	Exact outcome used	Unit	Direction indicating improvement	Handling of multiple outcomes
					outcomes were used
Sun et al. 2022	Technical accuracy	Average score / winning rate in simulated competition	score or %	Higher value = better	Selected or combined
Pak et al. 2020	Technical accuracy	Successful kick percentage	%	Higher value = better	Selected
Saremi et al. 2025	Endurance performance	Time to exhaustion / VO_2max / ventilatory threshold	s, $mL \cdot kg^{-1} \cdot min^{-1}$, or threshold value	Higher value = better	Selected or combined
Saremi et al. 2025	Explosive performance	Vertical jump height / relative average power	cm or W/kg	Higher value = better	Selected or combined
Delleli et al. 2023	Reaction agility	TSAT	s	Lower value = better; effect direction reversed	Selected
Delleli et al. 2023	FSKT-10s	FSKT-10s	number of kicks	Higher value = better	Selected
Delleli et al. 2023	FSKT-mult	FSKT-mult	number of kicks or decrement index	Higher value or lower decrement = better	Selected or direction reversed if required
Ouergui et al. 2022	Reaction agility	TSAT	s	Lower value = better; effect direction reversed	Selected
Ouergui et al. 2022	FSKT-10s	FSKT-10s	number of kicks	Higher value = better	Selected
Ouergui et al. 2022	FSKT-mult	FSKT-mult	number of kicks or decrement index	Higher value or lower decrement = better	Selected or direction reversed if required

Study	Meta-analysis domain	Exact outcome used	Unit	Direction indicating improvement	Handling of multiple outcomes
Ouergui et al. 2023	Reaction agility	TSAT	s	Lower value = better; effect direction reversed	Selected
Ouergui et al. 2023	FSKT-10s	FSKT-10s	number of kicks	Higher value = better	Selected
Ouergui et al. 2023	FSKT-mult	FSKT-mult	number of kicks or decrement index	Higher value or lower decrement = better	Selected or direction reversed if required
Nana et al. 2025	Reaction agility	Reaction agility / road agility test	s	Lower value = better; effect direction reversed	Selected
Cortez et al. 2017	Reaction agility	Rectus femoris reaction time / EMG-related reaction indicator	ms	Lower value = better; effect direction reversed	Selected
Delleli et al. 2024a	Explosive performance	Jump time or jump-related performance	s or cm	Direction adjusted according to the original metric	Selected
Delleli et al. 2024b	Reaction agility	TSAT	s	Lower value = better; effect direction reversed	Selected
Delleli et al. 2024b	FSKT-10s	FSKT-10s	number of kicks	Higher value = better	Selected
Delleli et al. 2024b	FSKT-mult	FSKT-mult	number of kicks or decrement index	Higher value or lower decrement = better	Selected or direction reversed if required
